# Supplementary material for: Temporal Shifts in MicroRNAs Signify the Inflammatory State of Primary Murine Microglial Cells
Source: Int J Mol Sci. 2025 Jun 13;26(12):5677. doi: 10.3390/ijms26125677 (PMC12193602; doi:10.3390/ijms26125677)
Supplement: Supplementary file 1 [file ijms-26-05677-s001.zip › ijms-3584768-supplementary.pdf]

Temporal shifts in microRNAs signify the inflammatory state of primary murine microglial cells

Keren Zohar[1], Elyad Lezmi[2], Fanny Reichert[3], Tsiona Eliyahu[1], Shlomo Rotshenker[3], Marta Weinstock[3], Michal Linial[1,#]

[1]Department of Biological Chemistry and [2]Department of Genetics, Faculty of Life Sciences, [3]Institute of Drug Research, School of Pharmacy, The Hebrew University of Jerusalem, Jerusalem, Israel

List of supplementary information

Supplementary **Figure S1.** Dendogram of miRNA-seq by experimental groups
Supplementary **Figure S2.** MA scatter plots for 3 h and 8 h relative to N.T.

Supplementary **Table S1.** Thresholds used for DEMs
Supplementary **Table S2.** Expression modules for all miRNA
Supplementary **Table S3.** List of 20 abundant miRNA, 8 h post activation
Supplementary **Table S4.** DEM for LPS relative to N.T. at 3 and 8 hours post activation
Supplementary **Table S5.** The normalized amounts of miRNA by TMM
Supplementary **Table S6.** DEM comparing LPS 3 h and 8 h.
Supplementary **Table S7.** List of miRNet2.0 mapping for 11 T-DEMs
Supplementary **Table S8.** DEM for LPS 8h with ladostigil relative to LPS 8h.

Supplementary Figures

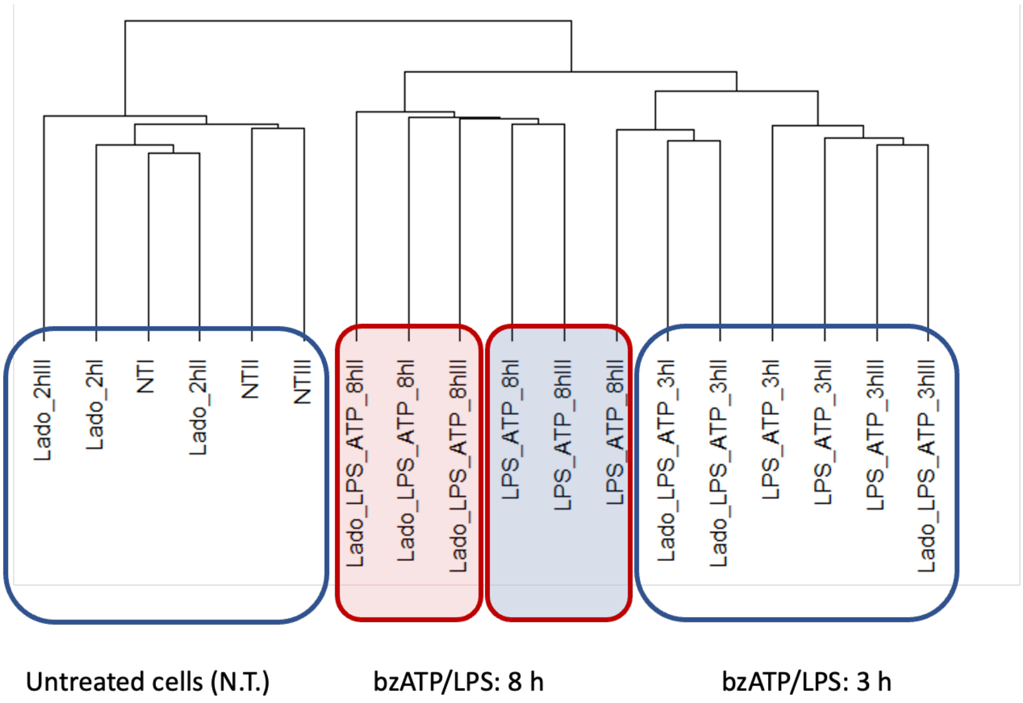

Figure S1. *Dendogram of the 18 tests and their control experiments. The dendogram used all mapped miRNAs (372). Each experiment is marked as I, II and III. The incubation of ladostigil (Lado) cased no effect as shown for N.T.) and similarly, the effect of shows that ladostigil in 3 h is negligible.*

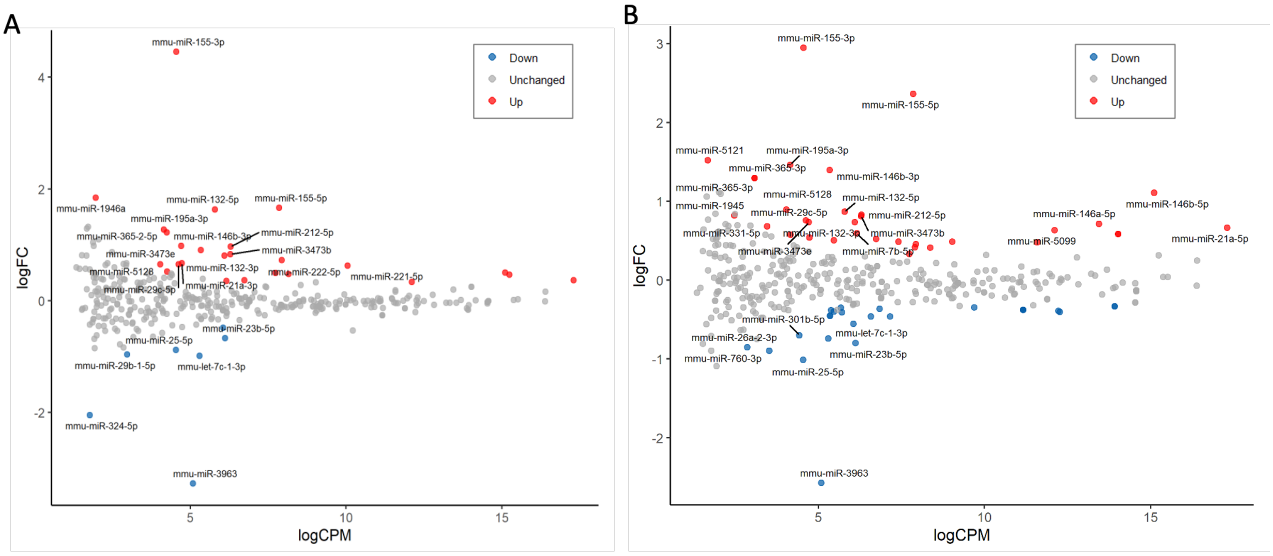

Figure S2. *The effect of microglial activation.* ***(A)*** *MA plots of the log2(FC) relative to the CPM (in log) for the 372 identified miRNAs in an activated setting (bzATP/LPS) 3 h post activation.* ***(B)*** *MA plots of the log2(FC) relative to the CPM (in log) miRNAs in an activated setting (bzATP/LPS) 8 h post activation relative to non-treated cells (N.T.).*
