# Supplementary material for: Temporal Shifts in MicroRNAs Signify the Inflammatory State of Primary Murine Microglial Cells
Source: Int J Mol Sci. 2025 Jun 13;26(12):5677. doi: 10.3390/ijms26125677 (PMC12193602; doi:10.3390/ijms26125677)
Supplement: Supplementary file 1 [file ijms-26-05677-s001.zip › MG-Revised2-final2-DOC-Supplemental Tables-S1-S8.pdf]

Table S1. Threshold for DEMs: Thresholds used for assigning DE genes for 9 clusters with distinct expression trends.

Trend	3 hours (logFC)	8 hours (logFC2)
↑ ↑	logFC > 0.33	logFC2 ≥ logFC+0.33
↑ ≈	logFC > 0.33	logFC2 ≤ logFC+0.33 & logFC2 ≥ logFC-0.33
↑ ↓	logFC > 0.33	logFC2 < logFC-0.33
≈ ↑	-0.5≤logFC ≤ 0.33	logFC2 > logFC+0.33
≈ ≈	-0.5≤logFC ≤ 0.33	logFC2 ≤logFC+0.33 &logFC2 ≥ logFC-0.33
≈ ↓	-0.5≤logFC ≤ 0.33	logFC2 < logFC-0.33
↓↑	logFC < -0.33	logFC2 > logFC+0.33
↓ ≈	logFC < -0.33	logFC2 ≤ logFC+0.33 & logFC2 ≥ logFC-0.33
↓↓	logFC < -0.33	logFC2 < logFC-0.33

↑ up, ↓ down, ≈ unchanged.

Table S2. Expression modules for all miRNA: Results of miRNA-seq for cells exposed with LPS

Combined trend The trend is define by the predetermne diffeential gene expression thresholds. There are three label called Up, Same and Down. The threshold are define according to Methods (see Table 1 for definition).

FC Fold change (FC) is based on the ratio of the mean (by TMM) of two experimental grops. The log base is 2. For example, down-down indicates that there is a substantial downregulation in gene expression at 3hrs relative to NT cells and at 8hrs relative to 3 hrs. All together there are 9 combined trends .

miRNA	logFC (0 to 3 hours of LPS)	logFC (0 to 8 hours of LPS)	Trend
mmu-miR-301a-3p	-0.427042988	-0.790106157	down_down
mmu-miR-760-3p	-0.333813605	-0.884596434	down_down
mmu-let-7b-3p	-0.479503152	-0.559465237	down_same
mmu-let-7c-1-3p	-0.969451953	-0.737778956	down_same
mmu-miR-181a-1-3p	-0.389760025	-0.237831922	down_same

mmu-miR-1839-3p	-0.442280762	-0.235881341	down_same
mmu-miR-199a-5p	-0.339265774	-0.451617485	down_same
mmu-miR-200a-5p	-0.466169682	-0.460581862	down_same
mmu-miR-23b-5p	-0.667891637	-0.799192187	down_same
mmu-miR-25-5p	-0.887080055	-1.01237102	down_same
mmu-miR-26a-2-3p	-0.842456382	-0.848193781	down_same
mmu-miR-26b-3p	-0.841687315	-1.097674402	down_same
mmu-miR-301a-5p	-0.349121878	-0.469389598	down_same
mmu-miR-301b-5p	-0.448445661	-0.714440737	down_same
mmu-miR-3057-5p	-0.538580272	-0.230526692	down_same
mmu-miR-324-3p	-0.350153031	-0.030914642	down_same
mmu-miR-3470a	-0.428291141	-0.260753931	down_same
mmu-miR-362-3p	-0.431282473	-0.426072427	down_same
mmu-miR-486a-5p	-0.4978041	-0.436900977	down_same
mmu-miR-486b-5p	-0.530071213	-0.438836392	down_same
mmu-miR-547-3p	-0.344248968	-0.437886775	down_same
mmu-miR-615-3p	-0.754624399	-0.618360974	down_same
mmu-miR-7676-3p	-0.335275554	-0.24735903	down_same
mmu-miR-8103	-0.5681391	-0.384355931	down_same
mmu-miR-15b-5p	-0.334463293	0.005719113	down_up
mmu-miR-16-2-3p	-0.621767274	0.642506188	down_up
mmu-miR-219a-1-3p	-0.526792596	0.148380665	down_up
mmu-miR-21b	-0.675209649	0.155215367	down_up
mmu-miR-223-3p	-0.472869315	0.115041678	down_up
mmu-miR-29b-1-5p	-0.966010684	-0.274337478	down_up
mmu-miR-324-5p	-2.045763738	-0.910234466	down_up
mmu-miR-351-3p	-0.509628672	-0.137148073	down_up
mmu-miR-3963	-3.195682795	-2.54881072	down_up
mmu-miR-500-3p	-0.36867436	0.035921919	down_up
mmu-miR-574-3p	-0.414572594	0.078214942	down_up

mmu-miR-664-3p	-0.649659161	0.08253498	down_up
mmu-miR-690	-0.406743992	0.092889066	down_up
mmu-miR-7015-3p	-0.37312717	-0.040464284	down_up
mmu-miR-7219-3p	-0.694866393	-0.353187924	down_up
mmu-miR-92a-3p	-0.532714835	-0.194731392	down_up
mmu-let-7d-3p	0.040934782	-0.320503924	same_down
mmu-miR-122-5p	0.217818001	-0.203367712	same_down
mmu-miR-188-5p	-0.014958061	-0.422728081	same_down
mmu-miR-1934-3p	-0.018776836	-0.586932348	same_down
mmu-miR-3079-5p	0.322837294	-0.172748918	same_down
mmu-miR-3103-3p	0.240638963	-0.36110201	same_down
mmu-miR-6960-5p	-0.114151399	-0.553038086	same_down
mmu-miR-6966-3p	-0.116583178	-0.805459637	same_down
mmu-miR-6994-3p	0.104793087	-0.386529451	same_down
mmu-miR-872-3p	0.314745402	-0.071484692	same_down
mmu-miR-877-3p	0.305593555	-0.094719088	same_down
mmu-miR-877-5p	0.103395516	-0.293993835	same_down
mmu-miR-98-3p	0.203916593	-0.225440439	same_down
mmu-let-7a-1-3p	-0.10203982	-0.180475953	same_same
mmu-let-7a-5p	-0.005107229	-0.128536312	same_same
mmu-let-7b-5p	-0.083644921	-0.402761309	same_same
mmu-let-7c-2-3p	-0.104360588	-0.181894859	same_same
mmu-let-7c-5p	-0.02516545	-0.285619321	same_same
mmu-let-7d-5p	-0.040398414	-0.183643046	same_same
mmu-let-7e-5p	0.028627835	0.043857021	same_same
mmu-let-7f-1-3p	-0.112877172	-0.184487	same_same
mmu-let-7f-2-3p	-0.172606354	-0.126666679	same_same
mmu-let-7f-5p	0.023139104	0.040644346	same_same
mmu-let-7g-5p	-0.039044405	0.058189259	same_same
mmu-let-7i-5p	0.163528737	0.247478548	same_same

mmu-let-7j	-0.003975158	0.171052379	same_same
mmu-miR-100-5p	-0.050187928	-0.109047247	same_same
mmu-miR-101a-3p	-0.15086051	-0.347340188	same_same
mmu-miR-101b-3p	-0.017117517	-0.057437868	same_same
mmu-miR-101c	0.091367621	-0.136727373	same_same
mmu-miR-103-3p	-0.111504778	-0.069970199	same_same
mmu-miR-106a-5p	-0.078567689	-0.111201736	same_same
mmu-miR-106b-3p	-0.133788727	-0.328796448	same_same
mmu-miR-106b-5p	-0.026485255	-0.234644391	same_same
mmu-miR-107-3p	-0.143244124	-0.126931969	same_same
mmu-miR-10a-3p	-0.273898539	-0.04379077	same_same
mmu-miR-10a-5p	-0.082003605	-0.090990421	same_same
mmu-miR-10b-5p	-0.053569696	-0.228998037	same_same
mmu-miR-1191a	0.200457315	0.384162288	same_same
mmu-miR-1198-5p	0.162162887	0.111283181	same_same
mmu-miR-125a-5p	-0.075688449	-0.054958325	same_same
mmu-miR-125b-2-3p	-0.16342983	-0.280457462	same_same
mmu-miR-125b-5p	-0.140066882	-0.238654556	same_same
mmu-miR-128-3p	0.001785614	-0.026223314	same_same
mmu-miR-1291	0.178209976	0.012509841	same_same
mmu-miR-130b-3p	-0.023127003	-0.154915348	same_same
mmu-miR-130b-5p	-0.063524526	-0.22172161	same_same
mmu-miR-139-5p	0.05346794	0.018367428	same_same
mmu-miR-140-3p	-0.125324206	-0.007110979	same_same
mmu-miR-140-5p	0.019430365	-0.045173726	same_same
mmu-miR-142a-3p	0.001083633	0.020905382	same_same
mmu-miR-142a-5p	-0.120234734	-0.127195812	same_same
mmu-miR-143-3p	0.093898057	-0.06328313	same_same
mmu-miR-148b-3p	-0.055793839	0.104452322	same_same
mmu-miR-148b-5p	0.00416127	0.192802836	same_same

mmu-miR-151-3p	-0.008705902	0.033734612	same_same
mmu-miR-151-5p	-0.090153512	0.101474981	same_same
mmu-miR-15a-5p	-0.182691956	-0.343074473	same_same
mmu-miR-15b-3p	-0.207755703	-0.135183525	same_same
mmu-miR-16-1-3p	0.153465915	-0.077180503	same_same
mmu-miR-16-5p	-0.03187532	-0.036159274	same_same
mmu-miR-17-3p	0.062444439	-0.130837299	same_same
mmu-miR-17-5p	-0.151966114	-0.034901643	same_same
mmu-miR-181a-2-3p	-0.262022882	-0.394693404	same_same
mmu-miR-181a-5p	-0.068012725	-0.154955294	same_same
mmu-miR-181b-5p	-0.118740655	-0.058447857	same_same
mmu-miR-181d-5p	-0.240275699	-0.35654778	same_same
mmu-miR-182-3p	-0.094353918	0.175242776	same_same
mmu-miR-182-5p	0.029676475	0.103076304	same_same
mmu-miR-183-5p	-0.029248392	-0.023017554	same_same
mmu-miR-1839-5p	-0.119427695	0.077707961	same_same
mmu-miR-1843a-5p	-0.062658052	-0.105378754	same_same
mmu-miR-1843b-3p	0.056475029	-0.148553123	same_same
mmu-miR-1843b-5p	-0.014739463	-0.059862931	same_same
mmu-miR-185-5p	-0.071441489	0.098234147	same_same
mmu-miR-186-5p	-0.095558695	-0.024759138	same_same
mmu-miR-18a-3p	0.04983404	0.2239904	same_same
mmu-miR-18a-5p	-0.102618645	-0.093997319	same_same
mmu-miR-191-5p	-0.041203459	0.000962504	same_same
mmu-miR-192-5p	0.239012259	0.218090102	same_same
mmu-miR-194-5p	0.03957562	-4.48E-05	same_same
mmu-miR-1943-5p	-0.050718269	-0.001706022	same_same
mmu-miR-1947-5p	0.157933655	0.162410641	same_same
mmu-miR-195a-5p	-0.001716082	-0.135143658	same_same
mmu-miR-1964-3p	0.04041823	0.126256831	same_same

mmu-miR-1981-3p	-0.223147818	0.012582293	same_same
mmu-miR-1981-5p	-0.012069347	-0.273715139	same_same
mmu-miR-1982-3p	0.074099085	0.180754725	same_same
mmu-miR-1983	0.287045194	0.560918169	same_same
mmu-miR-199a-3p	-0.005722521	-0.228843099	same_same
mmu-miR-199b-3p	-0.005722521	-0.228843099	same_same
mmu-miR-19a-3p	0.037146067	-0.053731457	same_same
mmu-miR-19b-3p	-0.094833802	-0.150655635	same_same
mmu-miR-200a-3p	-0.060224239	-0.204907372	same_same
mmu-miR-200b-3p	0.144757197	0.000972105	same_same
mmu-miR-200c-3p	-0.274321586	0.020519097	same_same
mmu-miR-20a-5p	-0.094909943	-0.005162556	same_same
mmu-miR-210-3p	-0.006526335	-0.152973234	same_same
mmu-miR-215-5p	0.125857259	0.271194173	same_same
mmu-miR-22-3p	0.188210292	0.477270938	same_same
mmu-miR-22-5p	0.253299293	0.486652697	same_same
mmu-miR-221-3p	0.102707867	0.286176864	same_same
mmu-miR-223-5p	0.170317116	-0.045620748	same_same
mmu-miR-23a-3p	-0.061900668	0.201017009	same_same
mmu-miR-23b-3p	-0.039163119	0.123163395	same_same
mmu-miR-24-1-5p	0.040532642	-0.020637913	same_same
mmu-miR-24-2-5p	0.042582722	-0.028208644	same_same
mmu-miR-24-3p	-0.007491497	-0.144022405	same_same
mmu-miR-25-3p	-0.11265435	-0.172927641	same_same
mmu-miR-26a-5p	-0.165083528	-0.333188931	same_same
mmu-miR-26b-5p	-0.129681525	-0.085335157	same_same
mmu-miR-27a-3p	-0.139565432	-0.100492607	same_same
mmu-miR-27a-5p	-0.180524958	-0.223772074	same_same
mmu-miR-27b-3p	-0.050363932	-0.039436687	same_same
mmu-miR-27b-5p	-0.258490666	-0.284708689	same_same

mmu-miR-28a-3p	-0.041348102	0.238380821	same_same
mmu-miR-28a-5p	-0.142231222	0.01858511	same_same
mmu-miR-28c	-0.077091785	0.053243909	same_same
mmu-miR-29a-3p	-0.045617576	-0.049311969	same_same
mmu-miR-29b-3p	0.062860268	0.182398122	same_same
mmu-miR-29c-3p	-0.005265029	0.233313088	same_same
mmu-miR-3066-5p	-0.090027256	0.030612427	same_same
mmu-miR-3068-3p	0.16173787	0.242845285	same_same
mmu-miR-3068-5p	0.17613422	0.001037227	same_same
mmu-miR-3074-5p	-0.020773217	-0.291018364	same_same
mmu-miR-30a-3p	0.001991079	-0.126107221	same_same
mmu-miR-30a-5p	-0.044762253	-0.107297454	same_same
mmu-miR-30b-3p	-0.219072227	0.032053677	same_same
mmu-miR-30b-5p	-0.026746491	0.144039484	same_same
mmu-miR-30c-1-3p	-0.194199228	-0.134801282	same_same
mmu-miR-30c-2-3p	-0.094652322	-0.103017497	same_same
mmu-miR-30c-5p	-0.014577106	-0.022575909	same_same
mmu-miR-30d-3p	-0.116513326	-0.002684602	same_same
mmu-miR-30d-5p	0.006388258	-0.031673071	same_same
mmu-miR-30e-3p	0.084338224	0.022717064	same_same
mmu-miR-30e-5p	-0.034190605	-0.041424072	same_same
mmu-miR-32-3p	-0.239022599	-0.33703298	same_same
mmu-miR-32-5p	-0.076102034	-0.140552302	same_same
mmu-miR-320-3p	0.004876472	0.017087596	same_same
mmu-miR-322-3p	0.119586841	0.303405338	same_same
mmu-miR-322-5p	0.300395705	0.236125527	same_same
mmu-miR-326-3p	-0.105407341	-0.241260344	same_same
mmu-miR-328-3p	-0.022599352	-0.180856572	same_same
mmu-miR-33-5p	0.224180322	0.043425717	same_same
mmu-miR-330-3p	0.056062522	0.234810979	same_same

mmu-miR-330-5p	-0.119585899	0.001576842	same_same
mmu-miR-338-3p	-0.114678354	-0.383663033	same_same
mmu-miR-338-5p	-0.295568968	-0.402904997	same_same
mmu-miR-339-3p	-0.084490318	-0.379404064	same_same
mmu-miR-339-5p	-0.083948495	-0.375358458	same_same
mmu-miR-340-3p	0.015085981	-0.122247728	same_same
mmu-miR-340-5p	-0.109933724	-0.106137649	same_same
mmu-miR-342-3p	0.175188065	-0.08475197	same_same
mmu-miR-345-3p	-0.102289857	0.135944416	same_same
mmu-miR-3470b	-0.124626094	-0.251590327	same_same
mmu-miR-3474	-0.125139545	-0.152787229	same_same
mmu-miR-34a-5p	0.03500455	0.15396014	same_same
mmu-miR-350-5p	0.246531014	0.15241989	same_same
mmu-miR-351-5p	0.275850562	0.217584265	same_same
mmu-miR-3535	0.005871228	0.240328093	same_same
mmu-miR-361-3p	0.130648644	0.411885885	same_same
mmu-miR-361-5p	0.105637488	0.256716783	same_same
mmu-miR-374b-5p	-0.049623534	0.12018593	same_same
mmu-miR-378a-3p	-0.207452941	-0.392502972	same_same
mmu-miR-378a-5p	-0.19944068	-0.284086704	same_same
mmu-miR-378b	0.035066455	0.062073778	same_same
mmu-miR-378c	-0.063109745	-0.185279677	same_same
mmu-miR-378d	-0.219054594	-0.231999017	same_same
mmu-miR-3968	-0.169860264	-0.456785929	same_same
mmu-miR-3970	0.028135721	0.168067991	same_same
mmu-miR-421-3p	0.072312056	0.199599318	same_same
mmu-miR-423-3p	0.044178453	0.077350667	same_same
mmu-miR-423-5p	0.02954053	-0.076302266	same_same
mmu-miR-425-3p	-0.013103461	0.056251698	same_same
mmu-miR-425-5p	0.000875621	0.068569745	same_same

mmu-miR-450a-5p	-0.018218545	-0.027480932	same_same
mmu-miR-450b-3p	0.183473886	0.142468501	same_same
mmu-miR-450b-5p	0.098656326	-0.0684871	same_same
mmu-miR-484	-0.005868979	0.018676791	same_same
mmu-miR-501-3p	-0.102733347	0.020359652	same_same
mmu-miR-503-3p	0.014051522	-0.050448854	same_same
mmu-miR-503-5p	0.031332198	0.306968151	same_same
mmu-miR-504-5p	-0.054810335	-0.366933771	same_same
mmu-miR-505-5p	0.044481623	-0.257149593	same_same
mmu-miR-5100	-0.068526938	-0.049454497	same_same
mmu-miR-5114	-0.243809172	-0.115079355	same_same
mmu-miR-5129-3p	0.24847197	0.31516849	same_same
mmu-miR-5134-3p	-0.140411091	-0.00057838	same_same
mmu-miR-532-5p	0.012012546	0.124911312	same_same
mmu-miR-542-3p	0.193419614	-0.064757303	same_same
mmu-miR-574-5p	-0.2996284	-0.298563506	same_same
mmu-miR-582-3p	-0.026353983	-0.025299268	same_same
mmu-miR-6412	-0.260636314	-0.509142758	same_same
mmu-miR-652-3p	-0.232831976	-0.165102332	same_same
mmu-miR-6539	-0.163700868	-0.134980992	same_same
mmu-miR-664-5p	-0.321247034	-0.339870417	same_same
mmu-miR-674-3p	0.086794353	0.047661015	same_same
mmu-miR-6899-3p	0.214077374	0.239947774	same_same
mmu-miR-6945-3p	0.176815001	0.348075142	same_same
mmu-miR-700-3p	-0.196222626	-0.195488507	same_same
mmu-miR-704	0.148622882	-0.064258729	same_same
mmu-miR-7043-3p	0.125052211	0.15369329	same_same
mmu-miR-7062-5p	0.115199216	0.0060511	same_same
mmu-miR-7118-3p	0.029002207	0.002420375	same_same
mmu-miR-744-5p	0.112152138	-0.035940375	same_same

mmu-miR-7670-3p	0.209014819	0.105867923	same_same
mmu-miR-8112	0.207365082	0.267346885	same_same
mmu-miR-872-5p	-0.077628825	-0.175868487	same_same
mmu-miR-9-3p	-0.164851587	-0.294403341	same_same
mmu-miR-9-5p	-0.259984617	-0.376133727	same_same
mmu-miR-92b-3p	0.019947021	-0.032792911	same_same
mmu-miR-93-5p	-0.075605885	-0.238777239	same_same
mmu-miR-96-5p	-0.082720143	-0.016490545	same_same
mmu-miR-98-5p	-0.005463515	0.010140341	same_same
mmu-miR-99a-5p	-0.014622357	-0.070567044	same_same
mmu-miR-99b-3p	0.328144525	0.488658206	same_same
mmu-miR-99b-5p	0.014136973	0.131172625	same_same
mmu-miR-1249-3p	-0.140163455	0.336968278	same_up
mmu-miR-146a-5p	0.194954865	0.714786333	same_up
mmu-miR-149-5p	0.260998858	0.66776232	same_up
mmu-miR-1945	0.21244557	0.804346378	same_up
mmu-miR-1949	-0.287861942	0.29369632	same_up
mmu-miR-21c	-0.157876981	0.452138649	same_up
mmu-miR-331-3p	-0.200932769	0.226725862	same_up
mmu-miR-331-5p	0.025483389	0.658062388	same_up
mmu-miR-342-5p	-0.293681546	0.051977321	same_up
mmu-miR-350-3p	-0.220173397	0.505793355	same_up
mmu-miR-362-5p	-0.234340676	0.267680741	same_up
mmu-miR-365-3p	-0.159400435	1.298148873	same_up
mmu-miR-429-3p	-0.316864833	0.60642218	same_up
mmu-miR-532-3p	0.143260671	0.527238225	same_up
mmu-miR-582-5p	-0.155700004	0.664821295	same_up
mmu-miR-6516-5p	-0.294791223	0.451720334	same_up
mmu-miR-671-3p	-0.074110232	0.415427672	same_up
mmu-miR-671-5p	-0.224775913	0.567089029	same_up

mmu-miR-674-5p	0.072134014	0.498788085	same_up
mmu-miR-6933-5p	0.027419594	0.457147399	same_up
mmu-miR-7059-5p	-0.268995861	0.417525604	same_up
mmu-miR-744-3p	0.096366891	0.5350658	same_up
mmu-miR-7648-3p	0.164036848	1.095747351	same_up
mmu-miR-7a-5p	0.245528138	0.584466023	same_up
mmu-miR-93-3p	-0.032160705	0.660728116	same_up
mmu-miR-1195	1.325826259	0.322785626	up_down
mmu-miR-1231-3p	1.305035214	0.760146697	up_down
mmu-miR-1306-3p	0.400658287	-0.032054	up_down
mmu-miR-1306-5p	0.349665931	-0.338075703	up_down
mmu-miR-132-5p	1.64535495	0.890645311	up_down
mmu-miR-139-3p	0.484175054	0.081982419	up_down
mmu-miR-155-3p	4.461859631	2.956034069	up_down
mmu-miR-1843a-3p	0.475608013	0.046518704	up_down
mmu-miR-1934-5p	0.386278549	0.028756387	up_down
mmu-miR-193a-5p	0.614800126	0.086145213	up_down
mmu-miR-1946a	1.852609354	0.61048176	up_down
mmu-miR-20a-3p	0.466354811	-0.131101175	up_down
mmu-miR-301b-3p	0.590935817	0.022230959	up_down
mmu-miR-3064-5p	0.393737663	-0.258019572	up_down
mmu-miR-3076-3p	0.442217609	0.106229542	up_down
mmu-miR-3082-3p	0.562148198	-0.113572145	up_down
mmu-miR-3473f	1.06936895	0.307434532	up_down
mmu-miR-365-2-5p	1.242952821	0.318469871	up_down
mmu-miR-455-5p	0.543209901	-0.702485746	up_down
mmu-miR-5126	0.783482685	0.352326398	up_down
mmu-miR-677-5p	0.638881922	0.008108252	up_down
mmu-miR-6948-3p	0.659792191	0.139362921	up_down
mmu-miR-6952-3p	0.671284612	-0.04959261	up_down

mmu-miR-7679-3p	0.604217822	-0.140961186	up_down
mmu-miR-92a-1-5p	0.451128189	-0.178550928	up_down
mmu-miR-935	0.334896555	-0.077796552	up_down
mmu-let-7e-3p	0.405280891	0.575915416	up_same
mmu-let-7i-3p	0.48966014	0.334438738	up_same
mmu-miR-1198-3p	0.862365955	0.800602754	up_same
mmu-miR-125a-3p	0.371819075	0.523651056	up_same
mmu-miR-125b-1-3p	0.498393356	0.329484827	up_same
mmu-miR-132-3p	0.809849662	0.742761592	up_same
mmu-miR-148a-3p	0.532573385	0.432793258	up_same
mmu-miR-191-3p	0.373167784	0.198186535	up_same
mmu-miR-1931	0.434969842	0.378048027	up_same
mmu-miR-195a-3p	1.2704303	1.437745961	up_same
mmu-miR-212-5p	0.969377405	0.823867534	up_same
mmu-miR-21a-3p	0.684652705	0.549180049	up_same
mmu-miR-21a-5p	0.362310797	0.665592671	up_same
mmu-miR-221-5p	0.62617813	0.317743809	up_same
mmu-miR-222-3p	0.46313649	0.313490112	up_same
mmu-miR-222-5p	0.726111426	0.457194739	up_same
mmu-miR-23a-5p	0.666007936	0.677759448	up_same
mmu-miR-29c-5p	0.641828119	0.753379866	up_same
mmu-miR-3098-3p	0.490629838	0.297106749	up_same
mmu-miR-3473b	0.837523277	0.794620638	up_same
mmu-miR-3473e	0.994430069	0.71955503	up_same
mmu-miR-3962	0.393372635	0.070371112	up_same
mmu-miR-5099	0.331503501	0.634124162	up_same
mmu-miR-5107-3p	1.094359309	1.049568633	up_same
mmu-miR-5128	0.649359166	0.875367291	up_same
mmu-miR-6911-3p	0.465995766	0.296492466	up_same
mmu-miR-6963-3p	0.42231128	0.440671562	up_same

mmu-miR-7667-5p	1.009145242	0.821029918	up_same
mmu-miR-7a-1-3p	0.393939567	0.441167535	up_same
mmu-miR-7b-5p	0.347919715	0.581533161	up_same
mmu-miR-8114	0.449980197	0.396872854	up_same
mmu-miR-146b-3p	0.916433887	1.410789597	up_up
mmu-miR-146b-5p	0.499749618	1.106029328	up_up
mmu-miR-155-5p	1.658136251	2.362221056	up_up
mmu-miR-29b-2-5p	0.353983926	0.720335893	up_up
mmu-miR-5121	0.79569012	1.536666874	up_up
mmu-miR-6240	0.590704917	1.062675372	up_up

Table S3. List of abundant (average CPM ≥100) DEMs 8 h post activation in the presence of bzATP/LPS

miRNA	FDR	Fold change	Ave. CPM	Trend
mmu-miR-155-5p	2.04E-15	5.14	231	Up
mmu-miR-146b-5p	3.29E-16	2.15	35,262.00	Up
mmu-miR-146a-5p	3.17E-07	1.64	11,147.50	Up
mmu-miR-21a-5p	3.06E-13	1.59	161,946.40	Up
mmu-miR-5099	8.14E-07	1.55	4,425.20	Up
mmu-miR-7a-5p	7.51E-09	1.5	16,738.00	Up
mmu-miR-125a-3p	2.87E-06	1.44	106.7	Up
mmu-miR-99b-3p	3.54E-06	1.4	523.7	Up
mmu-miR-22-5p	1.27E-04	1.4	169.7	Up
mmu-miR-22-3p	9.06E-08	1.39	3,072.80	Up
mmu-miR-222-5p	1.90E-05	1.37	243.8	Up
mmu-miR-671-3p	5.08E-03	1.33	238.8	Up
mmu-miR-361-3p	6.20E-05	1.33	330.7	Up

mmu-miR-26a-5p	2.87E-06	0.79	15,464.00	Down
mmu-miR-101a-3p	2.07E-05	0.79	825	Down
mmu-miR-504-5p	9.77E-03	0.78	114.8	Down
mmu-miR-9-5p	2.87E-06	0.77	2,290.40	Down
mmu-miR-378a-3p	1.47E-07	0.76	4,776.90	Down
mmu-let-7b-5p	1.95E-03	0.76	4,927.10	Down
mmu-miR-3968	7.01E-03	0.73	142.6	Down

Table S4. DEM for LPS 3h relative to N.T

miRNA	precursor	logFC	logCPM	F	PValue	FDR	color
mmu-let-7c-1-3p	mmu-let-7c-1	-0.96945	5.295865	37.70492	2.01E-06	5.75E-05	Down
mmu-miR-23b-5p	mmu-mir-23b	-0.66789	6.111998	37.18416	2.24E-06	5.94E-05	Down
mmu-miR-25-5p	mmu-mir-25	-0.88708	4.537792	23.20855	5.95E-05	0.001054	Down
mmu-let-7b-3p	mmu-let-7b	-0.4795	6.054437	12.39896	0.001671	0.016415	Down
mmu-miR-324-5p	mmu-mir-324	-2.04576	1.763745	12.15544	0.001831	0.017469	Down
mmu-miR-3963	mmu-mir-3963	-3.19568	5.082475	10.83541	0.002961	0.026865	Down
mmu-miR-29b-1-5p	mmu-mir-29b-1	-0.96601	2.971918	10.45276	0.003426	0.030344	Down
mmu-miR-301a-5p	mmu-mir-301a	-0.34912	6.574308	9.040241	0.005934	0.048636	Down
mmu-miR-155-3p	mmu-mir-155	4.46186	4.551287	361.3782	2.43E-16	9.05E-14	Up
mmu-miR-155-5p	mmu-mir-155	1.658136	7.851463	219.1879	6.80E-14	1.27E-11	Up
mmu-miR-132-5p	mmu-mir-132	1.645355	5.783253	140.4731	9.12E-12	1.13E-09	Up
mmu-miR-221-5p	mmu-mir-221	0.626178	10.05333	131.2367	1.88E-11	1.75E-09	Up
mmu-miR-146b-5p	mmu-mir-146b	0.49975	15.10582	119.1822	5.18E-11	3.85E-09	Up
mmu-miR-222-5p	mmu-mir-222	0.726111	7.929817	105.9547	1.74E-10	1.08E-08	Up
mmu-miR-212-5p	mmu-mir-212	0.969377	6.290504	101.6714	2.66E-10	1.41E-08	Up
mmu-miR-21a-5p	mmu-mir-21a	0.362311	17.30516	87.32793	1.21E-09	5.64E-08	Up
mmu-miR-222-3p	mmu-mir-222	0.463136	15.24541	57.59962	5.98E-08	2.47E-06	Up
mmu-miR-146b-3p	mmu-mir-146b	0.916434	5.335017	51.39237	1.62E-07	6.01E-06	Up

mmu-miR-132-3p	mmu-mir-132	0.80985	6.094335	48.45234	2.66E-07	9.01E-06	Up
mmu-miR-125b-1-3p	mmu-mir-125b-1	0.498393	7.732373	42.71206	7.52E-07	2.33E-05	Up
mmu-miR-195a-3p	mmu-mir-195a	1.27043	4.148304	33.27765	5.15E-06	0.000128	Up
mmu-miR-125a-3p	mmu-mir-125a	0.371819	6.737397	26.07202	2.82E-05	0.000655	Up
mmu-miR-365-2-5p	mmu-mir-365-2	1.242953	4.241474	25.50702	3.26E-05	0.000712	Up
mmu-miR-3473e	mmu-mir-3473e	0.99443	4.705864	21.44262	9.67E-05	0.001498	Up
mmu-miR-3473b	mmu-mir-3473b	0.837523	6.285275	20.28542	0.000134	0.001998	Up
mmu-miR-21a-3p	mmu-mir-21a	0.684653	4.722027	19.23277	0.000183	0.002425	Up
mmu-miR-1946a	mmu-mir-1946a	1.852609	1.956379	18.19864	0.000208	0.00267	Up
mmu-miR-5099	mmu-mir-5099	0.331504	12.11151	17.31326	0.000326	0.004047	Up
mmu-miR-139-3p	mmu-mir-139	0.484175	8.159731	16.4023	0.000434	0.005213	Up
mmu-miR-29c-5p	mmu-mir-29c	0.641828	4.617987	13.29221	0.00122	0.013753	Up
mmu-miR-5128	mmu-mir-5128	0.649359	4.032887	12.38934	0.001677	0.016415	Up
mmu-miR-7b-5p	mmu-mir-7b	0.34792	6.153375	11.02553	0.002757	0.02564	Up
mmu-miR-148a-3p	mmu-mir-148a	0.532573	4.247586	10.06063	0.003976	0.034393	Up

DEM for LPS 8h relative to N.T

miRNA	precursor	logFC	logCPM	F	PValue	FDR	color
mmu-miR-378a-3p	mmu-mir-378a	-0.3925	12.22186	78.18967	3.55E-09	1.47E-07	Down
mmu-miR-26a-5p	mmu-mir-26a-1	-0.33319	13.91613	53.40055	1.16E-07	2.87E-06	Down
mmu-miR-26a-5p	mmu-mir-26a-2	-0.33308	13.91663	53.33592	1.17E-07	2.87E-06	Down
mmu-miR-9-5p	mmu-mir-9-1	-0.37676	11.16135	52.33023	1.38E-07	2.87E-06	Down
mmu-miR-9-5p	mmu-mir-9-2	-0.37676	11.16136	52.32447	1.38E-07	2.87E-06	Down
mmu-miR-9-5p	mmu-mir-9-3	-0.37613	11.15954	52.30264	1.39E-07	2.87E-06	Down
mmu-miR-23b-5p	mmu-mir-23b	-0.79919	6.111998	50.76865	1.79E-07	3.51E-06	Down
mmu-miR-101a-3p	mmu-mir-101a	-0.34734	9.688251	39.76894	1.33E-06	2.07E-05	Down
mmu-miR-25-5p	mmu-mir-25	-1.01237	4.537792	28.57644	1.52E-05	0.000188	Down

mmu-let-7c-1-3p	mmu-let-7c-1	-0.73778	5.295865	22.86286	6.53E-05	0.000675	Down
mmu-let-7b-5p	mmu-let-7b	-0.40276	12.26654	18.93946	0.000199	0.001949	Down
mmu-let-7b-3p	mmu-let-7b	-0.55947	6.054437	16.28303	0.000451	0.003904	Down
mmu-miR-301a-5p	mmu-mir-301a	-0.46939	6.574308	15.71118	0.000542	0.004481	Down
mmu-miR-301b-5p	mmu-mir-301b	-0.71444	4.422846	15.34483	0.000611	0.004836	Down
mmu-miR-338-5p	mmu-mir-338	-0.4029	5.700257	14.53771	0.000798	0.006058	Down
mmu-miR-3968	mmu-mir-3968	-0.45679	7.155682	14.0435	0.000943	0.007012	Down
mmu-miR-504-5p	mmu-mir-504	-0.36693	6.842803	12.91332	0.001392	0.009773	Down
mmu-miR-199a-5p	mmu-mir-199a-1	-0.45162	5.346109	11.71809	0.002136	0.01435	Down
mmu-miR-199a-5p	mmu-mir-199a-2	-0.45162	5.345397	11.57251	0.002252	0.014506	Down
mmu-miR-760-3p	mmu-mir-760	-0.8846	3.519034	11.28259	0.002507	0.01581	Down
mmu-miR-664-5p	mmu-mir-664	-0.33987	5.675005	10.02523	0.00403	0.022712	Down
mmu-miR-181a-2-3p	mmu-mir-181a-2	-0.39469	5.46081	8.566429	0.007185	0.034711	Down
mmu-miR-339-3p	mmu-mir-339	-0.3794	5.373847	8.315122	0.007963	0.037497	Down
mmu-miR-3963	mmu-mir-3963	-2.54881	5.082475	8.033041	0.00895	0.041616	Down
mmu-miR-146b-5p	mmu-mir-146b	1.106029	15.10582	573.2474	8.83E-19	3.29E-16	Up
mmu-miR-155-5p	mmu-mir-155	2.362221	7.851463	464.3991	1.10E-17	2.04E-15	Up
mmu-miR-21a-5p	mmu-mir-21a	0.665593	17.30516	292.9268	2.47E-15	3.06E-13	Up
mmu-miR-146b-3p	mmu-mir-146b	1.41079	5.335017	136.3113	1.26E-11	1.17E-09	Up
mmu-miR-7a-5p	mmu-mir-7a-1	0.584466	14.03084	110.4953	1.13E-10	7.51E-09	Up
mmu-miR-7a-5p	mmu-mir-7a-2	0.583739	14.02166	109.7977	1.21E-10	7.51E-09	Up
mmu-miR-155-3p	mmu-mir-155	2.956034	4.551287	99.70822	3.44E-10	1.83E-08	Up
mmu-miR-22-3p	mmu-mir-22	0.477271	11.58534	83.20031	1.95E-09	9.06E-08	Up
mmu-miR-146a-5p	mmu-mir-146a	0.714786	13.44443	70.98926	8.88E-09	3.17E-07	Up
mmu-miR-212-5p	mmu-mir-212	0.823868	6.290504	70.57764	9.38E-09	3.17E-07	Up
mmu-miR-5099	mmu-mir-5099	0.634124	12.11151	63.12864	2.62E-08	8.14E-07	Up
mmu-miR-125a-3p	mmu-mir-125a	0.523651	6.737397	52.4838	1.35E-07	2.87E-06	Up
mmu-miR-99b-3p	mmu-mir-99b	0.488658	9.032577	50.41248	1.90E-07	3.54E-06	Up
mmu-miR-195a-3p	mmu-mir-195a	1.437746	4.148304	44.05679	5.87E-07	1.04E-05	Up
mmu-miR-222-5p	mmu-mir-222	0.457195	7.929817	40.6201	1.12E-06	1.90E-05	Up

mmu-miR-132-3p	mmu-mir-132	0.742762	6.094335	39.738	1.34E-06	2.07E-05	Up
mmu-miR-132-5p	mmu-mir-132	0.890645	5.783253	34.53051	3.91E-06	5.82E-05	Up
mmu-miR-361-3p	mmu-mir-361	0.411886	8.369534	34.0581	4.33E-06	6.20E-05	Up
mmu-miR-7b-5p	mmu-mir-7b	0.581533	6.153375	31.89682	7.00E-06	9.30E-05	Up
mmu-miR-22-5p	mmu-mir-22	0.486653	7.406645	30.39076	9.88E-06	0.000127	Up
mmu-miR-5128	mmu-mir-5128	0.875367	4.032887	23.70273	5.22E-05	0.000554	Up
mmu-miR-29c-5p	mmu-mir-29c	0.75338	4.617987	18.62693	0.000219	0.002086	Up
mmu-miR-3473b	mmu-mir-3473b	0.794621	6.285275	17.80048	0.000281	0.002548	Up
mmu-miR-365-3p	mmu-mir-365-1	1.298149	3.072524	15.7644	0.000534	0.004481	Up
mmu-miR-365-3p	mmu-mir-365-2	1.298149	3.076104	15.65252	0.000554	0.004481	Up
mmu-miR-671-3p	mmu-mir-671	0.415428	7.899618	15.12917	0.000656	0.005081	Up
mmu-miR-21a-3p	mmu-mir-21a	0.54918	4.722027	11.74211	0.002117	0.01435	Up
mmu-miR-3473e	mmu-mir-3473e	0.719555	4.705864	10.21233	0.00375	0.021643	Up
mmu-miR-5121	mmu-mir-5121	1.536667	1.662237	9.314935	0.005356	0.028514	Up
mmu-let-7e-3p	mmu-let-7e	0.575915	4.161528	8.726019	0.006735	0.033404	Up
mmu-miR-674-5p	mmu-mir-674	0.498788	5.462166	8.427895	0.007603	0.03626	Up
mmu-miR-331-5p	mmu-mir-331	0.658062	3.454201	7.942675	0.009295	0.04269	Up
mmu-miR-1945	mmu-mir-1945	0.804346	2.46002	7.666915	0.010466	0.046463	Up

Table S5. Normalized amounts of miRNA by TMM

**miRNA**	**precursor**	**NT**	**lado 2h**	**ATP 3h**	**ATP/LPS 3h**	**Lado ATP/LPS 3G**	**ATP 8h**	**ATP/LPS 8h**	**Lado ATP/LPS 8h**
mmu-let-7a-5p	mmu-let-7a-1	25553.39	25044.51	25174.29	25474.87	24370.11	23797.35	23377.5	22814.44
mmu-let-7a-1-3p	mmu-let-7a-1	515.424	514.6953	506.8791	479.9397	478.1847	473.778	455.2038	499.2611
mmu-let-7a-5p	mmu-let-7a-2	25533.13	25003.75	25137.75	25442.53	24337.02	23770.46	23356.02	22784.45
mmu-let-7b-5p	mmu-let-7b	5457.864	5341.013	5291.393	5149.311	4631.581	5116.862	4127.822	4297.166
mmu-let-7b-3p	mmu-let-7b	82.64066	82.63006	72.73042	58.90378	46.25944	71.78051	56.48662	57.12358
mmu-let-7c-5p	mmu-let-7c-1	25353.99	25371.91	25157.07	24914.73	22948.14	24163.77	20799.83	21905.64

mmu-let-7c-1-3p	mmu-let-7c-1	47.05241	57.1867	49.5774	23.61717	25.57949	46.28063	28.20016	33.08503
mmu-let-7c-5p	mmu-let-7c-2	25389.91	25426.04	25209.48	24964.6	22989.16	24206.25	20845.97	21934.36
mmu-let-7c-2-3p	mmu-let-7c-2	515.8341	514.3645	506.8646	479.5223	477.713	473.249	455.1051	498.6089
mmu-let-7d-5p	mmu-let-7d	12629.24	12375.31	12252.77	12280.33	11980.15	11932.67	11118.91	10693.18
mmu-let-7d-3p	mmu-let-7d	2964.895	3118.715	2849.791	3048.492	2478.775	2905.646	2374.38	2950.909
mmu-let-7e-5p	mmu-let-7e	2208.401	2120.636	2142.645	2252.264	2136.179	2084.086	2275.182	2402.169
mmu-let-7e-3p	mmu-let-7e	14.00894	13.37924	14.57129	18.66425	21.29041	16.14417	20.8294	21.08668
mmu-let-7f-5p	mmu-let-7f-1	42801.82	41362.87	44559.54	43195.34	44077.14	41116.79	43976.12	39780.75
mmu-let-7f-1-3p	mmu-let-7f-1	52.88709	49.2327	44.1256	48.47135	41.67974	43.74851	46.96043	45.82215
mmu-let-7f-5p	mmu-let-7f-2	46852.84	45809.38	48926.88	47611	48255.22	45220.81	48191.56	43993.33
mmu-let-7f-2-3p	mmu-let-7f-2	6.632374	6.3234	5.886703	6.049489	5.677509	7.744697	5.951968	7.064594
mmu-let-7g-5p	mmu-let-7g	10371.54	10288.07	10674.78	10095.64	10765.17	10061.26	10797.86	9695.022
mmu-let-7i-5p	mmu-let-7i	79745.28	81924.15	80921.53	89316.48	90927.67	80498.84	94669.19	96240.41
mmu-let-7i-3p	mmu-let-7i	10.08107	14.9205	10.03857	14.03007	9.492206	13.52747	12.2979	12.49223
mmu-let-7j	mmu-let-7j	2143.135	2108.419	2190.197	2137.64	2253.507	2182.696	2413.73	2279.212
mmu-miR-100-5p	mmu-mir-100	1885.8	1889.455	1850.964	1820.913	1793.093	1764.839	1749.047	1756.471
mmu-miR-101a-3p	mmu-mir-101a	919.2854	897.2998	906.0861	828.6378	815.6971	776.3507	723.0896	728.2217
mmu-miR-101b-3p	mmu-mir-101b	669.4729	664.9816	657.9757	661.673	648.8882	632.149	643.4889	634.0142
mmu-miR-101c	mmu-mir-101c	17.02275	17.3024	17.13579	18.23011	15.24611	13.89451	15.42819	15.36045
mmu-miR-103-3p	mmu-mir-103-1	558.5966	557.0571	578.9058	518.0817	590.8304	566.7091	530.9113	521.032
mmu-miR-103-3p	mmu-mir-103-2	571.7663	576.7571	595.2274	529.1334	607.261	586.949	547.9939	532.9565
mmu-miR-106a-5p	mmu-mir-106a	6.350961	5.884888	5.039741	6.089808	6.606738	5.530171	6.040215	4.182757
mmu-miR-106b-5p	mmu-mir-106b	57.52428	64.90454	60.95443	56.82731	59.17187	58.82323	48.65266	47.19159
mmu-miR-106b-3p	mmu-mir-106b	646.6835	691.8349	635.1819	588.9237	584.1765	657.5984	515.6058	553.2086
mmu-miR-107-3p	mmu-mir-107	73.99577	74.94301	67.69781	67.45401	73.59171	77.66536	66.91673	63.91019
mmu-miR-10a-5p	mmu-mir-10a	16420.51	15999.09	16254.17	15513.21	15089.71	16020.18	15417.91	15233.74
mmu-miR-10a-3p	mmu-mir-10a	77.101	65.22681	76.45977	63.71369	72.24984	75.8404	74.25115	60.76248
mmu-miR-10b-5p	mmu-mir-10b	51.44299	55.23191	52.39177	50.13935	45.4293	46.37387	44.43097	49.02604
mmu-miR-1191a	mmu-mir-1191a	16.7093	17.39085	17.40615	19.61371	21.07542	19.51527	21.79227	20.98218
mmu-miR-1195	mmu-mir-1195	1.450435	1.737254	2.276609	3.692445	3.460609	4.14138	1.929805	3.587139
mmu-miR-1198-5p	mmu-mir-1198	2856.961	2800.84	2899.608	3195.191	3157.448	3047.271	3086.915	3540.174

mmu-miR-1198-3p	mmu-mir-1198	3.071971	5.778239	3.754372	5.663786	5.587387	6.009713	5.705323	5.843982
mmu-miR-122-5p	mmu-mir-122	11.10306	10.32685	8.747035	12.54342	7.884888	7.19609	9.589512	7.928555
mmu-miR-1231-3p	mmu-mir-1231	1.482238	3.457554	2.521876	3.517356	3.865401	1.868675	2.530683	2.626986
mmu-miR-1249-3p	mmu-mir-1249	25.39438	25.56402	24.64877	23.06244	23.02793	27.68179	32.9688	22.98095
mmu-miR-125a-5p	mmu-mir-125a	7618.675	7140.141	7097.759	7228.566	6958.058	7188.556	7332.533	7393.422
mmu-miR-125a-3p	mmu-mir-125a	91.84803	79.00678	92.42611	118.3758	113.0606	95.54502	131.573	128.8508
mmu-miR-125b-5p	mmu-mir-125b-1	4166.584	4103.348	3914.081	3790.325	3706.776	3754.213	3530.527	3482.694
mmu-miR-125b-1-3p	mmu-mir-125b-1	179.8614	164.681	208.9499	253.8328	222.7773	204.6523	226.5891	236.8324
mmu-miR-125b-5p	mmu-mir-125b-2	4155.981	4081.291	3894.205	3771.289	3680.105	3728.642	3510.8	3466.624
mmu-miR-125b-2-3p	mmu-mir-125b-2	248.7179	245.6354	239.0908	222.0283	231.2877	229.2864	205.4289	199.6238
mmu-miR-128-3p	mmu-mir-128-1	4820.192	5063.902	5069.683	4845.143	4696.419	4956.099	4734.24	5083.011
mmu-miR-128-3p	mmu-mir-128-2	3615.932	3766.5	3736.968	3619.526	3494.52	3725.381	3535.868	3823.163
mmu-miR-1291	mmu-mir-1291	3.538917	4.120668	2.638011	4.312827	4.195797	2.350488	3.904416	3.297304
mmu-miR-1306-5p	mmu-mir-1306	6.8636	4.050821	5.888855	8.608892	5.79476	6.572716	5.343633	5.620779
mmu-miR-1306-3p	mmu-mir-1306	12.45167	11.07875	10.86637	15.90938	12.38375	13.18197	12.02908	11.41754
mmu-miR-130b-5p	mmu-mir-130b	1618.336	1660.811	1681.023	1548.271	1528.27	1615.954	1387.69	1548.207
mmu-miR-130b-3p	mmu-mir-130b	32.68972	29.87646	34.9952	31.77119	33.47747	35.14595	28.80512	32.98077
mmu-miR-132-5p	mmu-mir-132	31.19056	34.96824	39.88405	96.35755	77.65815	39.59473	56.36072	60.696
mmu-miR-132-3p	mmu-mir-132	48.90422	54.83397	55.00588	85.38572	86.18187	57.76126	81.27907	73.70113
mmu-miR-139-5p	mmu-mir-139	4872.114	5019.656	5056.075	5055.267	4927.192	5048.528	4934.649	5296.06
mmu-miR-139-3p	mmu-mir-139	260.3638	246.7	276.1763	362.3459	312.5013	275.9364	275.5104	275.5108
mmu-miR-140-5p	mmu-mir-140	399.2816	399.1108	374.2435	405.0372	392.3498	394.6035	387.3623	399.981
mmu-miR-140-3p	mmu-mir-140	829.6642	844.3439	801.0822	760.8073	839.7066	801.7741	825.7128	806.0672
mmu-miR-142a-5p	mmu-mir-142a	1933.255	1891.049	1894.008	1778.803	1792.783	1685.697	1769.81	1622.234
mmu-miR-142a-3p	mmu-mir-142a	1649.273	1608.929	1615.251	1650.386	1656.566	1634.113	1672.065	1686.271
mmu-miR-143-3p	mmu-mir-143	57.36132	56.35516	59.47511	60.81641	54.62939	59.63032	53.77984	57.92156
mmu-miR-146a-5p	mmu-mir-146a	9700.915	8994.34	9450.452	11104.99	11666.15	9039.057	15920.33	13299.58
mmu-miR-146b-5p	mmu-mir-146b	24583.08	24044.57	26320.31	34759.32	34553.8	34732.36	52915.58	50187.08
mmu-miR-146b-3p	mmu-mir-146b	22.11127	23.74125	29.30951	41.31612	51.14764	36.59665	57.90728	58.70073
mmu-miR-148a-3p	mmu-mir-148a	14.76823	16.5958	20.01655	21.1435	15.9711	19.4602	20.04815	20.50456
mmu-miR-148b-5p	mmu-mir-148b	32.88012	40.8543	41.0818	33.51197	36.63829	33.38859	38.18568	36.67469

mmu-miR-148b-3p	mmu-mir-148b	872.9394	828.0913	850.2862	839.9966	890.2559	916.5023	937.7579	942.9755
mmu-miR-149-5p	mmu-mir-149	3.631081	5.691652	6.228283	4.637313	5.1839	6.186807	6.115812	5.947044
mmu-miR-151-5p	mmu-mir-151	39.3546	42.9524	40.03271	36.92252	40.76875	40.06746	41.67991	40.69217
mmu-miR-151-3p	mmu-mir-151	5550.388	5708.616	5775.307	5516.159	5442.98	6048.919	5682.708	6545.502
mmu-miR-155-5p	mmu-mir-155	93.92669	93.10884	102.0908	297.6419	281.7495	107.7502	484.3819	387.17
mmu-miR-155-3p	mmu-mir-155	2.890649	2.359167	5.074048	62.83324	57.59176	4.730038	22.08031	25.45465
mmu-miR-15a-5p	mmu-mir-15a	14.3532	10.36607	13.18374	12.4746	13.00421	11.27368	11.28861	12.18764
mmu-miR-15b-5p	mmu-mir-15b	61.10214	58.60664	63.79702	48.33315	58.19498	63.89401	60.95001	55.5504
mmu-miR-15b-3p	mmu-mir-15b	53.00999	51.69066	51.42912	46.24858	53.74631	48.91125	47.67931	46.77057
mmu-miR-16-5p	mmu-mir-16-1	599.0521	615.473	620.0001	585.8859	593.8757	572.3588	583.3732	546.6277
mmu-miR-16-1-3p	mmu-mir-16-1	129.5675	129.103	129.341	144.1081	146.6734	123.6149	122.621	139.2882
mmu-miR-16-5p	mmu-mir-16-2	597.3937	614.4096	618.854	584.3156	592.3413	569.8115	581.7307	543.8467
mmu-miR-16-2-3p	mmu-mir-16-2	3.008793	4.368357	3.347525	1.935021	3.874103	3.325786	4.504468	2.822837
mmu-miR-17-5p	mmu-mir-17	608.492	596.6142	599.6771	547.2973	593.9821	606.1049	592.7693	563.9364
mmu-miR-17-3p	mmu-mir-17	31.9981	38.19269	34.688	33.35751	34.09499	30.44119	29.4135	29.00244
mmu-miR-181a-5p	mmu-mir-181a-1	1072.167	1090.969	1059.55	1022.382	1009.293	965.6812	962.0231	934.4839
mmu-miR-181a-1-3p	mmu-mir-181a-1	7.978224	9.486628	8.265854	6.067081	6.285392	6.894373	6.901437	5.876062
mmu-miR-181a-5p	mmu-mir-181a-2	1072.077	1090.969	1059.386	1022.382	1009.293	965.6045	961.8103	934.3934
mmu-miR-181a-2-3p	mmu-mir-181a-2	49.5118	50.68582	46.06496	40.68896	37.96635	49.06912	37.54265	37.25003
mmu-miR-181b-5p	mmu-mir-181b-1	313.8959	318.912	324.675	288.8986	315.9273	311.9945	300.9476	295.8271
mmu-miR-181b-5p	mmu-mir-181b-2	316.9937	323.2534	328.591	293.9121	318.777	316.2458	302.9401	298.495
mmu-miR-181d-5p	mmu-mir-181d	8.872507	9.6973	9.39165	7.352723	7.654943	6.949891	6.550775	5.043499
mmu-miR-182-5p	mmu-mir-182	2716.473	2869.428	2899.733	2773.286	2824.441	2882.068	2918.944	3042.621
mmu-miR-182-3p	mmu-mir-182	10.65894	11.72543	11.58405	9.514136	12.1016	12.48193	12.24876	14.46757
mmu-miR-183-5p	mmu-mir-183	3047.783	3002.26	2973.919	2986.007	2877.608	3058.792	3000.168	2995.544
mmu-miR-1839-5p	mmu-mir-1839	252.2689	236.5862	257.9721	232.0494	264.8543	225.4208	264.996	220.2264
mmu-miR-1839-3p	mmu-mir-1839	9.840495	8.544766	8.59808	7.531491	7.482889	6.900781	8.601448	8.603221
mmu-miR-1843a-5p	mmu-mir-1843a	343.8872	351.8695	341.21	329.3976	326.1095	328.525	320.2173	308.8947
mmu-miR-1843a-3p	mmu-mir-1843a	17.68486	20.05862	23.65039	24.82781	20.57648	19.14115	18.50105	20.21926
mmu-miR-1843b-5p	mmu-mir-1843b	369.4411	372.3769	369.0075	366.0126	356.794	344.0247	355.5329	349.1835
mmu-miR-1843b-3p	mmu-mir-1843b	128.89	122.605	127.7693	133.8648	116.5433	125.6189	116.1493	139.2579

mmu-miR-185-5p	mmu-mir-185	79.41793	83.50572	90.07607	75.26914	82.9597	78.82653	84.22228	68.1391
mmu-miR-186-5p	mmu-mir-186	383.3623	373.0318	352.2125	358.8295	377.7989	364.8141	376.6521	378.2977
mmu-miR-188-5p	mmu-mir-188	8.582315	9.726985	9.529612	8.529253	6.720127	6.427289	6.405102	8.274235
mmu-miR-18a-5p	mmu-mir-18a	34.25246	29.51004	35.74667	31.64306	32.87883	34.7035	31.08488	35.35511
mmu-miR-18a-3p	mmu-mir-18a	32.93708	32.52085	32.39602	33.9433	34.08283	33.15117	38.17957	33.79406
mmu-miR-191-5p	mmu-mir-191	5185.074	5050.901	4867.6	5038.437	5333.152	5088.793	5186.869	5081.621
mmu-miR-191-3p	mmu-mir-191	36.44855	36.30082	41.71892	47.93655	43.3184	40.86829	42.50255	45.36622
mmu-miR-192-5p	mmu-mir-192	157.3887	172.3399	163.9403	185.6175	180.5788	157.9769	183.0363	185.6173
mmu-miR-1931	mmu-mir-1931	15.91935	10.31433	16.55504	21.17618	22.42517	20.11679	21.48075	33.57148
mmu-miR-1934-5p	mmu-mir-1934	6.546268	5.459969	6.809032	8.482664	6.097981	7.524217	6.707042	7.942479
mmu-miR-1934-3p	mmu-mir-1934	5.466357	6.062424	6.721705	5.087402	6.424704	6.858918	3.341561	6.437393
mmu-miR-193a-5p	mmu-mir-193a	6.138074	4.278141	7.35296	9.677956	8.743497	5.735191	6.536415	8.617211
mmu-miR-194-5p	mmu-mir-194-1	27.73806	32.33805	30.44095	28.61578	28.03683	28.9093	27.73263	26.76621
mmu-miR-194-5p	mmu-mir-194-2	29.41953	33.05293	32.17111	30.51482	30.59464	30.88295	30.61935	29.12247
mmu-miR-1943-5p	mmu-mir-1943	3.447606	3.642727	4.369508	3.327639	3.487253	2.99993	3.416244	3.141101
mmu-miR-1945	mmu-mir-1945	3.515894	4.336992	4.983828	4.070708	5.309506	3.1253	6.316525	8.766543
mmu-miR-1946a	mmu-mir-1946a	1.734333	0.296007	3.215424	6.289134	7.14065	2.12468	2.645564	3.402573
mmu-miR-1947-5p	mmu-mir-1947	13.77772	15.7983	15.84122	15.53673	14.06245	14.36494	15.89456	15.4881
mmu-miR-1949	mmu-mir-1949	2.328297	3.076213	3.462843	1.903515	8.04041	3.72455	2.80009	3.058401
mmu-miR-195a-5p	mmu-mir-195a	4.312099	2.58334	3.540886	4.25378	3.075273	3.572724	3.885569	1.975836
mmu-miR-195a-3p	mmu-mir-195a	9.961836	7.91466	11.02015	24.11762	23.51391	9.148218	27.74407	26.28154
mmu-miR-1964-3p	mmu-mir-1964	250.5038	256.6812	277.7227	256.9953	265.8567	274.0236	274.082	311.5618
mmu-miR-1981-5p	mmu-mir-1981	355.4849	373.7003	384.6168	352.4743	363.2248	377.0169	294.8493	373.5331
mmu-miR-1981-3p	mmu-mir-1981	17.66075	17.83199	17.04995	15.20446	16.17091	16.87484	17.59856	17.28775
mmu-miR-1982-3p	mmu-mir-1982	5.904648	6.043613	5.125283	6.11584	5.754063	6.283449	6.492524	6.507428
mmu-miR-1983	mmu-mir-1983	8.379081	8.817649	9.99728	10.61388	9.786351	10.4569	12.63338	9.433576
mmu-miR-199a-5p	mmu-mir-199a-1	48.30039	46.53879	44.2993	38.61066	39.58083	36.52596	35.29377	31.41132
mmu-miR-199a-3p	mmu-mir-199a-1	123.1677	120.9582	127.0597	123.5664	123.3486	113.0393	104.964	100.3382
mmu-miR-199a-5p	mmu-mir-199a-2	48.30039	46.46497	44.2993	38.53222	39.58083	36.52596	35.29377	31.41132
mmu-miR-199a-3p	mmu-mir-199a-2	123.2571	121.5794	127.1101	124.5688	124.4936	114.2914	105.7852	100.9338
mmu-miR-199b-3p	mmu-mir-199b	123.1677	120.9582	127.0597	123.5664	123.3486	113.0393	104.964	100.3382

mmu-miR-19a-3p	mmu-mir-19a	95.56053	100.5086	102.253	98.20127	110.256	100.0504	92.14331	94.09694
mmu-miR-19b-3p	mmu-mir-19b-1	118.6379	121.5228	109.587	110.6319	109.6404	109.664	107.2999	107.3157
mmu-miR-19b-3p	mmu-mir-19b-2	118.2304	121.2707	109.4469	110.8498	109.5048	109.8532	106.5783	105.9399
mmu-miR-200a-5p	mmu-mir-200a	4.091616	4.110099	4.694673	3.048879	2.714136	3.726813	2.961399	4.385479
mmu-miR-200a-3p	mmu-mir-200a	29.65471	32.19394	28.43782	28.30651	26.29804	25.34824	25.35787	25.80107
mmu-miR-200b-3p	mmu-mir-200b	46.02562	44.90748	47.20098	50.77748	50.27961	48.93844	45.66612	48.86505
mmu-miR-200c-3p	mmu-mir-200c	39.78928	33.51457	40.06509	32.77156	36.73702	40.58097	40.26482	40.665
mmu-miR-20a-5p	mmu-mir-20a	566.2361	527.3875	562.9909	529.6352	593.1814	571.2609	562.5982	537.6222
mmu-miR-20a-3p	mmu-mir-20a	2.674946	4.520178	2.644991	3.764406	3.614163	2.990131	2.350407	4.191835
mmu-miR-210-3p	mmu-mir-210	414.5317	432.2984	411.5994	413.2264	386.3661	360.974	372.5919	368.5531
mmu-miR-212-5p	mmu-mir-212	55.54462	53.70628	54.19388	108.4371	96.90232	62.37657	98.55786	94.17989
mmu-miR-215-5p	mmu-mir-215	3.284432	3.831779	4.677003	3.811505	3.923318	4.671518	4.164295	4.312987
mmu-miR-219a-1-3p	mmu-mir-219a-1	5.46832	6.433288	5.41347	3.693614	6.481664	5.007966	6.131208	7.834754
mmu-miR-21a-5p	mmu-mir-21a	127589.9	131006.2	135454	164015.2	173457	153083.1	202386.5	208578.5
mmu-miR-21a-3p	mmu-mir-21a	21.01757	18.33107	22.39897	33.36905	28.12064	21.23099	30.41292	33.08061
mmu-miR-21b	mmu-mir-21b	8.944486	9.274795	7.776287	5.768423	8.584592	8.34429	9.896703	8.643118
mmu-miR-21c	mmu-mir-21c	4.837774	4.771013	4.163522	4.309319	5.125261	4.420785	6.479544	4.477256
mmu-miR-22-5p	mmu-mir-22	145.7319	145.7996	158.2751	173.7991	169.4562	148.8888	204.1418	208.5421
mmu-miR-22-3p	mmu-mir-22	2721.6	2737.02	2700.34	3100.978	3112.758	2863.942	3788.625	3554.194
mmu-miR-221-5p	mmu-mir-221	861.2559	835.3037	961.1318	1329.478	1371.54	887.8552	1073.851	1176.387
mmu-miR-221-3p	mmu-mir-221	6514.333	6468.173	6908.292	6996.258	7959.706	6778.613	7942.04	6836.428
mmu-miR-222-5p	mmu-mir-222	191.4425	189.399	210.0615	315.9461	302.62	195.959	262.6839	279.5677
mmu-miR-222-3p	mmu-mir-222	33212.28	34010.65	34135.34	45783.44	43456.07	35879.98	41273.93	42999.19
mmu-miR-223-5p	mmu-mir-223	78.09215	81.18086	74.79201	87.88878	85.95177	72.81937	76.19467	79.94879
mmu-miR-223-3p	mmu-mir-223	29.09174	28.82871	25.64996	21.01363	26.77087	22.31329	31.34189	22.10136
mmu-miR-23a-5p	mmu-mir-23a	4.030922	9.668905	7.514907	6.476681	8.083769	8.473455	6.593871	6.542478
mmu-miR-23a-3p	mmu-mir-23a	530.1661	514.4948	566.0991	509.2713	577.1326	545.6402	608.2965	489.9839
mmu-miR-23b-5p	mmu-mir-23b	90.07454	75.21168	74.41959	56.43927	52.54235	73.20419	51.84425	76.39633
mmu-miR-23b-3p	mmu-mir-23b	1207.476	1296.277	1288.995	1176.281	1337.241	1292.981	1314.735	1164.779
mmu-miR-24-1-5p	mmu-mir-24-1	157.4917	178.4387	172.9846	162.8659	167.3029	161.3025	156.0576	156.9581
mmu-miR-24-3p	mmu-mir-24-1	24673.96	25580.64	25444.64	24546.05	24947.12	24291.63	22329.69	23701.46

mmu-miR-24-3p	mmu-mir-24-2	24674.03	25580.72	25444.72	24546.13	24947.27	24291.95	22329.96	23701.46
mmu-miR-24-2-5p	mmu-mir-24-2	2032.21	2119.017	2133.805	2092.379	2080.018	2068.467	1993.107	2125.758
mmu-miR-25-5p	mmu-mir-25	30.62665	30.77297	27.01381	16.62525	17.36474	24.17994	15.17509	17.92648
mmu-miR-25-3p	mmu-mir-25	2009.951	1959.055	1929.068	1858.544	1949.019	1996.41	1782.502	1937.899
mmu-miR-26a-5p	mmu-mir-26a-1	17295.36	17619.94	16357.14	15424.78	15168.73	14818.35	13728.41	13251.33
mmu-miR-26a-5p	mmu-mir-26a-2	17299.42	17627.37	16362.68	15431.53	15173.16	14825.39	13732.68	13255
mmu-miR-26a-2-3p	mmu-mir-26a-2	8.31155	8.609481	8.51655	4.654736	3.644506	8.561426	4.556522	6.999222
mmu-miR-26b-5p	mmu-mir-26b	2478.093	2374.051	2463.756	2265.105	2369.093	2254.029	2333.909	2099.807
mmu-miR-26b-3p	mmu-mir-26b	4.922012	3.835016	2.787933	2.708766	3.405112	3.301989	2.352028	2.508041
mmu-miR-27a-5p	mmu-mir-27a	550.6085	482.2403	640.9679	485.4786	481.162	542.0447	472.1653	599.8665
mmu-miR-27a-3p	mmu-mir-27a	15967.21	15013.96	14868.95	14493.53	14922.66	14508.98	14891.71	15482.29
mmu-miR-27b-5p	mmu-mir-27b	179.7738	177.8891	182.013	150.1911	150.2507	158.9475	148.6545	188.6282
mmu-miR-27b-3p	mmu-mir-27b	24331.38	24033.86	24717.15	23496.58	24617.92	23232.89	23675.03	23176.79
mmu-miR-28a-5p	mmu-mir-28a	217.8326	221.1604	193.3536	197.1259	218.6014	218.7874	220.0615	224.7051
mmu-miR-28a-3p	mmu-mir-28a	197.1842	185.2049	184.2275	191.5838	202.7381	207.6029	232.0627	221.4448
mmu-miR-28c	mmu-mir-28c	15.02047	15.18622	12.88196	14.18563	14.04355	17.02356	15.92476	16.90343
mmu-miR-29a-3p	mmu-mir-29a	3206.198	3244.712	3141.246	3106.686	3113.075	2991.384	3098.319	2885.949
mmu-miR-29b-1-5p	mmu-mir-29b-1	8.958281	7.874016	7.452831	4.587249	6.873681	7.171173	7.30951	8.773597
mmu-miR-29b-3p	mmu-mir-29b-1	105.0069	102.7702	114.6483	109.6697	107.9651	106.5317	118.2913	102.6574
mmu-miR-29b-3p	mmu-mir-29b-2	105.0069	102.7702	114.8127	109.6697	107.9651	106.5317	118.2913	102.6574
mmu-miR-29b-2-5p	mmu-mir-29b-2	3.305918	2.212263	3.757194	4.400007	3.301146	5.24998	5.369141	5.82224
mmu-miR-29c-5p	mmu-mir-29c	17.57502	19.64526	24.44494	27.62245	24.51976	21.1004	29.78184	28.77382
mmu-miR-29c-3p	mmu-mir-29c	16.32411	16.29487	18.29567	16.21197	18.17539	15.44261	19.32791	16.27276
mmu-miR-301a-5p	mmu-mir-301a	110.1163	108.9084	99.54789	86.62819	88.16657	100.6503	80.15012	83.61337
mmu-miR-301a-3p	mmu-mir-301a	9.396382	10.21407	9.79761	7.311268	7.838814	9.97928	5.704738	5.764685
mmu-miR-301b-5p	mmu-mir-301b	23.75531	25.21037	24.1947	17.52632	17.55875	23.78054	14.71417	21.42349
mmu-miR-301b-3p	mmu-mir-301b	4.285738	6.407055	5.452232	6.508017	4.811477	5.403674	4.415096	5.373746
mmu-miR-3057-5p	mmu-mir-3057	4.464861	4.296005	4.190178	3.059657	5.434206	4.642456	3.637634	4.922282
mmu-miR-3064-5p	mmu-mir-3064	6.127757	7.86815	7.890421	7.98868	5.865242	5.805432	5.548502	7.219601
mmu-miR-3066-5p	mmu-mir-3066	3.568756	3.169184	3.12721	3.188191	4.113471	3.977888	3.33811	2.798125
mmu-miR-3068-5p	mmu-mir-3068	53.55074	58.19713	59.23513	59.74621	57.79377	56.4201	52.87695	61.31183

mmu-miR-3068-3p	mmu-mir-3068	14.61748	15.70739	15.41707	16.87928	17.20856	16.67957	18.0014	17.57071
mmu-miR-3074-5p	mmu-mir-3074-1	7.991167	8.177659	6.792698	7.944395	7.437772	8.041888	6.334667	8.324736
mmu-miR-3074-5p	mmu-mir-3074-2	7.426188	7.77375	6.792698	7.845232	7.191538	7.870464	6.334667	8.206986
mmu-miR-3076-3p	mmu-mir-3076	3.876433	5.149942	5.648416	5.168142	5.512833	5.701596	4.278622	5.520108
mmu-miR-3079-5p	mmu-mir-3079	2.782632	3.278552	4.202803	3.545523	3.032714	4.467633	2.452549	2.360916
mmu-miR-3082-3p	mmu-mir-3082	1.655301	2.588472	1.790232	2.529033	2.592602	2.359945	1.59961	2.349632
mmu-miR-3098-3p	mmu-mir-3098	2.299311	4.354855	3.359182	3.264457	2.863618	3.515735	3.035166	3.077936
mmu-miR-30a-5p	mmu-mir-30a	3323.759	3405.736	3262.214	3222.455	3206.265	3188.812	3085.606	3077.003
mmu-miR-30a-3p	mmu-mir-30a	913.9236	936.0321	888.4609	915.0252	900.3692	903.9282	837.762	873.7185
mmu-miR-30b-5p	mmu-mir-30b	67.1495	72.13624	69.87655	66.50401	66.05683	65.41549	73.96423	56.71208
mmu-miR-30b-3p	mmu-mir-30b	76.09702	67.79919	69.86022	65.29451	71.47716	70.2477	78.03372	77.40325
mmu-miR-30c-5p	mmu-mir-30c-1	2147.926	2268.868	2254.045	2127.112	2261.62	2119.009	2113.894	1962.945
mmu-miR-30c-1-3p	mmu-mir-30c-1	14.24126	11.62464	13.42887	12.17097	12.60187	10.66011	12.65269	11.99942
mmu-miR-30c-5p	mmu-mir-30c-2	2148.063	2269.267	2254.18	2127.033	2261.448	2119.192	2113.894	1962.903
mmu-miR-30c-2-3p	mmu-mir-30c-2	67.37983	69.29973	57.29295	62.25782	62.56844	64.97197	62.6648	63.29252
mmu-miR-30d-5p	mmu-mir-30d	18217.3	19455	18847.77	18298.33	18646.23	17690	17822.32	18261.92
mmu-miR-30d-3p	mmu-mir-30d	25.04167	29.50616	31.59398	23.30869	23.15668	25.83083	25.3379	29.68234
mmu-miR-30e-5p	mmu-mir-30e	812.755	881.2585	817.7574	793.5338	796.4391	832.4112	789.8446	801.9255
mmu-miR-30e-3p	mmu-mir-30e	525.2095	541.3469	538.5025	556.7738	570.7702	528.6762	533.5218	578.0963
mmu-miR-3103-3p	mmu-mir-3103	3.507968	5.920224	4.968013	4.038862	4.085922	5.521104	2.838699	4.336753
mmu-miR-32-5p	mmu-mir-32	248.7703	242.2252	249.5716	235.7788	243.7059	244.5803	225.2524	227.671
mmu-miR-32-3p	mmu-mir-32	13.72275	13.78004	15.22645	11.53668	12.37765	16.74982	10.77551	13.99801
mmu-miR-320-3p	mmu-mir-320	1166.414	1081.699	1132.078	1170.031	1178.033	1059.26	1180.017	1110.18
mmu-miR-322-5p	mmu-mir-322	14.3033	13.71878	15.75552	17.64821	15.95798	16.84006	17.10652	15.1699
mmu-miR-322-3p	mmu-mir-322	78.11626	69.59088	77.26799	84.81404	91.33077	86.60749	95.98367	87.80645
mmu-miR-324-5p	mmu-mir-324	4.711514	2.528119	2.39742	1.100604	3.271735	3.842719	2.588813	2.572899
mmu-miR-324-3p	mmu-mir-324	7.326334	7.602042	4.604605	5.884009	5.372217	6.993286	7.11546	6.227866
mmu-miR-326-3p	mmu-mir-326	65.53329	53.33058	56.63773	60.82206	60.52492	63.82983	55.05997	63.10525
mmu-miR-328-3p	mmu-mir-328	2005.658	2007.755	1934.58	1974.122	1846.285	1945.751	1768.77	1733.095
mmu-miR-33-5p	mmu-mir-33	5.838654	7.01348	4.542745	6.815115	6.872029	5.66415	5.976097	5.960536
mmu-miR-330-5p	mmu-mir-330	272.7687	277.0258	272.4384	251.038	263.5171	281.8251	273.6053	317.1355

mmu-miR-330-3p	mmu-mir-330	136.894	152.4678	150.7662	141.9357	149.0501	160.6042	161.1346	154.8782
mmu-miR-331-5p	mmu-mir-331	8.096368	10.59045	10.69714	8.256967	7.884166	11.94505	13.15558	14.41909
mmu-miR-331-3p	mmu-mir-331	13.29035	12.43268	15.17581	11.70767	13.55335	14.71838	15.89235	14.88599
mmu-miR-338-5p	mmu-mir-338	61.83517	49.74594	54.00267	50.27704	48.36577	52.65812	46.49173	48.29767
mmu-miR-338-3p	mmu-mir-338	16.75916	14.63834	14.97331	16.01065	15.28308	16.14224	13.02286	13.94128
mmu-miR-339-5p	mmu-mir-339	51.35646	48.25409	52.96822	48.62588	42.73359	49.75975	39.53907	39.98925
mmu-miR-339-3p	mmu-mir-339	43.52698	46.03526	46.93576	40.99348	39.38789	41.03778	33.39431	35.67811
mmu-miR-340-5p	mmu-mir-340	550.1838	554.553	555.8018	510.4673	524.4205	511.7946	511.0513	504.1862
mmu-miR-340-3p	mmu-mir-340	464.2208	486.7623	502.3979	469.141	455.9928	480.2566	426.9242	473.8668
mmu-miR-342-5p	mmu-mir-342	14.45644	15.63099	13.37651	11.32062	10.08436	16.59198	14.86433	18.14698
mmu-miR-342-3p	mmu-mir-342	8.70576	8.7267	10.86236	10.0431	10.00096	10.87798	8.068005	8.875464
mmu-miR-345-3p	mmu-mir-345	54.81628	58.66695	52.33103	50.9957	54.70394	62.91831	59.75274	54.06595
mmu-miR-3470a	mmu-mir-3470a	4.268017	2.539635	5.005137	3.0401	4.198032	5.307762	3.481277	5.709153
mmu-miR-3470b	mmu-mir-3470b	9.551488	8.757118	5.830791	8.835385	8.384595	10.66458	8.045032	10.40375
mmu-miR-3473b	mmu-mir-3473b	50.98798	46.70558	64.09436	90.3625	88.26198	72.48953	89.84071	122.1016
mmu-miR-3473e	mmu-mir-3473e	17.07079	14.35946	20.65017	33.42091	31.01456	23.60459	28.50626	38.95853
mmu-miR-3473f	mmu-mir-3473f	2.297016	2.431519	2.984636	4.525067	2.269578	3.175149	2.794929	5.475796
mmu-miR-3474	mmu-mir-3474	16.94462	16.54727	18.64913	15.2389	16.9159	14.40952	15.41857	17.50837
mmu-miR-34a-5p	mmu-mir-34a	91.01351	95.75254	96.36888	94.00407	94.59062	96.1348	101.1492	82.4386
mmu-miR-350-5p	mmu-mir-350	14.44255	15.98174	14.17632	16.84651	18.76759	15.51018	15.93842	16.32376
mmu-miR-350-3p	mmu-mir-350	2.722175	3.0206	2.486602	2.437479	4.542671	3.610114	3.987591	2.804747
mmu-miR-351-5p	mmu-mir-351	337.4149	323.494	340.7121	408.9585	407.8737	365.3257	392.0012	408.1575
mmu-miR-351-3p	mmu-mir-351	7.302554	4.048835	5.967785	5.152719	4.518447	4.629204	6.900057	5.280957
mmu-miR-3535	mmu-mir-3535	32.03492	28.88404	31.272	32.77565	48.89742	39.47066	37.41219	31.44246
mmu-miR-361-5p	mmu-mir-361	39.97187	42.10971	44.27301	43.21496	41.04242	41.76127	47.42484	45.62555
mmu-miR-361-3p	mmu-mir-361	286.13	299.7841	314.9519	312.8956	330.7209	335.4452	381.4644	382.0543
mmu-miR-362-5p	mmu-mir-362	19.09967	17.02042	17.80067	16.1714	18.59237	17.79836	22.5077	17.42037
mmu-miR-362-3p	mmu-mir-362	4.359659	3.597341	1.859959	3.264627	2.263669	2.460336	2.892794	2.124222
mmu-miR-365-3p	mmu-mir-365-1	5.613582	6.917827	6.983685	4.992679	9.163247	8.081542	13.66086	8.453889
mmu-miR-365-3p	mmu-mir-365-2	5.613582	7.066931	6.983685	4.992679	9.163247	8.081542	13.66086	8.453889
mmu-miR-365-2-5p	mmu-mir-365-2	11.83632	13.44918	17.08322	27.38818	21.82581	14.90227	14.89467	28.12828

mmu-miR-374b-5p	mmu-mir-374b	99.80738	104.2652	113.2105	97.63689	95.88264	91.79764	108.7266	87.6486
mmu-miR-378a-5p	mmu-mir-378a	77.76067	83.2955	70.37193	67.20971	61.59639	79.27815	62.98768	63.02675
mmu-miR-378a-3p	mmu-mir-378a	5312.643	5355.168	5125.289	4601.329	4775.168	5026.345	4047.498	3966.369
mmu-miR-378b	mmu-mir-378b	93.18803	101.5252	95.89089	95.7881	90.44729	96.47795	97.20817	86.84922
mmu-miR-378c	mmu-mir-378c	722.367	756.3221	724.2828	692.2687	683.5358	713.0358	635.8726	617.0438
mmu-miR-378d	mmu-mir-378d	560.6015	607.315	539.9848	482.4477	507.5514	538.2013	478.3212	416.0224
mmu-miR-3962	mmu-mir-3962	3.723576	3.480855	3.010405	4.880263	5.184805	4.244827	3.752636	2.819435
mmu-miR-3963	mmu-mir-3963	131.0168	12.51633	14.08654	13.38152	11.42657	11.92614	22.30533	60.99896
mmu-miR-3968	mmu-mir-3968	162.4744	141.7664	157.4546	144.1329	145.6583	143.4455	117.7117	122.8727
mmu-miR-3970	mmu-mir-3970	13.77251	15.18375	13.03716	13.92726	15.82756	13.18729	15.73508	15.84028
mmu-miR-421-3p	mmu-mir-421	24.71407	33.01738	31.53221	26.14616	29.0813	24.83962	28.46864	28.0445
mmu-miR-423-5p	mmu-mir-423	1213.951	1231.583	1163.634	1237.596	1117.207	1248.148	1152.846	1290.874
mmu-miR-423-3p	mmu-mir-423	3851.521	3972.087	3762.216	3971.24	3934.893	4100.504	4064.48	4185.666
mmu-miR-425-5p	mmu-mir-425	482.9779	485.5232	499.1482	483.8116	504.02	524.7547	506.7726	512.1711
mmu-miR-425-3p	mmu-mir-425	24.72153	22.36441	26.83805	24.44982	28.07398	23.58035	25.69806	22.85633
mmu-miR-429-3p	mmu-mir-429	3.912588	4.475526	6.576541	3.120704	4.765986	2.346693	6.22025	2.982691
mmu-miR-450a-5p	mmu-mir-450a-1	121.0897	125.3555	116.2851	119.9649	111.2888	110.4445	119.4094	126.4341
mmu-miR-450a-5p	mmu-mir-450a-2	121.2263	125.3555	116.2851	120.1272	111.2888	110.4445	119.4094	126.4341
mmu-miR-450b-5p	mmu-mir-450b	29.39431	30.0911	28.20028	31.3891	35.03462	30.1146	28.15879	28.22548
mmu-miR-450b-3p	mmu-mir-450b	10.76296	9.300332	11.72232	12.45772	11.51148	11.33329	11.46045	10.5771
mmu-miR-455-5p	mmu-mir-455	2.998145	3.482321	3.196267	4.519762	2.762977	1.872074	1.773778	2.503627
mmu-miR-484	mmu-mir-484	1492.311	1577.795	1456.173	1486.228	1478.421	1581.884	1511.211	1526.059
mmu-miR-486a-5p	mmu-mir-486a	11.26069	9.616364	7.672777	7.889822	8.57126	11.52103	7.840868	9.767723
mmu-miR-486b-5p	mmu-mir-486b	11.7336	10.20001	8.403751	8.042388	8.410865	11.88654	8.188409	10.14253
mmu-miR-500-3p	mmu-mir-500	3.629118	4.590759	2.92204	2.67065	2.804606	2.278374	3.683941	2.155988
mmu-miR-501-3p	mmu-mir-501	265.2519	238.4467	255.2942	246.2391	266.9601	264.7428	268.6345	269.3605
mmu-miR-503-5p	mmu-mir-503	34.48273	37.46858	36.59548	34.99714	41.28937	39.66831	42.09214	42.78904
mmu-miR-503-3p	mmu-mir-503	47.6301	47.4026	40.78677	47.6775	45.45811	50.43599	45.96085	49.81826
mmu-miR-504-5p	mmu-mir-504	116.9974	127.7192	117.3218	113.3067	116.49	114.8858	91.20064	116.7224
mmu-miR-505-5p	mmu-mir-505	19.63585	18.97481	20.34913	19.90943	15.67603	16.34018	16.26656	15.54495
mmu-miR-5099	mmu-mir-5099	3368.733	3568.064	4193.109	4239.131	4176.841	4143.667	5229.961	6481.855

mmu-miR-5100	mmu-mir-5100	4.088895	4.111351	4.107609	3.755627	2.969072	4.756098	3.955884	3.513701
mmu-miR-5107-3p	mmu-mir-5107	2.433498	4.389495	2.655832	5.209427	3.491325	2.525311	5.401674	3.220463
mmu-miR-5114	mmu-mir-5114	24.32235	23.24441	24.12355	20.70357	23.14247	23.5566	23.28244	21.70582
mmu-miR-5121	mmu-mir-5121	1.518724	2.134779	3.34188	2.731663	3.000136	1.380462	4.401531	2.307776
mmu-miR-5126	mmu-mir-5126	1.747465	1.915309	2.10374	3.209054	1.567874	2.412407	2.321237	4.012879
mmu-miR-5128	mmu-mir-5128	11.35191	10.22115	11.05357	17.82385	17.91838	13.88771	21.34263	25.17098
mmu-miR-5129-3p	mmu-mir-5129	9.365623	9.447932	7.713394	11.34613	9.451483	9.202998	12.31954	9.897769
mmu-miR-5134-3p	mmu-mir-5134	4.588069	5.739756	5.373524	4.126246	4.289224	5.61815	4.822509	6.034222
mmu-miR-532-5p	mmu-mir-532	1006.287	1018.63	1015.058	1015.009	1052.476	1078.701	1098.074	1099.696
mmu-miR-532-3p	mmu-mir-532	2.601451	2.505123	3.009886	3.026982	3.682993	5.303228	3.849496	2.410838
mmu-miR-542-3p	mmu-mir-542	18.49401	19.44002	19.37348	21.46185	18.39223	22.95095	17.63189	20.52605
mmu-miR-547-3p	mmu-mir-547	7.113069	6.228481	6.207856	5.817488	4.2798	4.264487	5.106726	5.381114
mmu-miR-574-5p	mmu-mir-574	13.34397	11.95184	11.33908	10.88613	7.807776	16.69468	10.74357	7.874468
mmu-miR-574-3p	mmu-mir-574	14.16887	15.15046	12.48121	10.64538	11.68316	13.65693	14.84707	9.374891
mmu-miR-582-5p	mmu-mir-582	2.472896	2.803544	2.92954	2.293692	1.325712	1.878871	3.782511	1.450502
mmu-miR-582-3p	mmu-mir-582	81.05196	79.35034	85.86013	80.39334	82.30686	74.89804	80.08824	73.59365
mmu-miR-615-3p	mmu-mir-615	7.385064	5.950123	7.463082	4.196072	6.000481	8.069473	4.745411	5.797463
mmu-miR-6240	mmu-mir-6240	2.078687	1.22251	0.752434	3.005289	2.367635	2.998009	4.279417	4.014139
mmu-miR-6412	mmu-mir-6412	5.449982	3.999264	5.23095	4.415955	4.669791	5.09974	3.817669	4.875828
mmu-miR-6516-5p	mmu-mir-6516	4.460081	5.180054	3.383464	3.493289	4.081293	3.464011	5.980133	3.369795
mmu-miR-652-3p	mmu-mir-652	161.8836	156.3581	148.8113	137.6986	145.4613	142.5889	144.1407	132.6189
mmu-miR-6539	mmu-mir-6539	11.51021	9.871207	9.628898	9.924871	8.828196	10.5003	10.43463	9.721518
mmu-miR-664-5p	mmu-mir-664	60.04396	47.97464	56.70188	47.66513	47.68147	54.64841	46.92222	42.6351
mmu-miR-664-3p	mmu-mir-664	16.06595	11.7593	13.0235	10.21041	12.43964	13.28394	16.99827	13.39899
mmu-miR-671-5p	mmu-mir-671	9.780419	9.336187	12.90402	8.517475	9.631518	12.98635	14.32961	13.47387
mmu-miR-671-3p	mmu-mir-671	202.7448	231.6314	220.5124	192.2876	203.2411	256.0004	271.0153	333.3541
mmu-miR-674-5p	mmu-mir-674	37.10349	34.31556	42.22548	39.07512	46.34019	45.71342	52.7954	50.45788
mmu-miR-674-3p	mmu-mir-674	3957.192	4088.856	4126.059	4201.769	4015.598	4272.857	4091.294	4686.213
mmu-miR-677-5p	mmu-mir-677	4.742889	5.89451	6.701581	7.602113	10.17682	6.87738	5.235083	5.99016
mmu-miR-6899-3p	mmu-mir-6899	4.138513	2.787323	5.329566	4.779761	3.852626	4.242961	5.013903	5.210987
mmu-miR-690	mmu-mir-690	35.66879	26.29857	34.31553	26.35313	56.08484	33.60852	37.53403	35.22388

mmu-miR-6911-3p	mmu-mir-6911	5.371708	6.185813	6.553594	7.200191	6.744934	4.858807	6.554467	4.882699
mmu-miR-6933-5p	mmu-mir-6933	4.18889	3.637809	3.1878	4.358757	6.450974	3.846514	5.646367	5.795072
mmu-miR-6945-3p	mmu-mir-6945	6.58046	5.060636	5.725643	7.148957	6.272802	4.859203	8.587914	6.481521
mmu-miR-6948-3p	mmu-mir-6948	3.15654	5.814522	4.160851	4.792218	4.598752	4.094587	3.133151	4.933632
mmu-miR-6952-3p	mmu-mir-6952	3.216475	5.967382	5.521441	5.101485	3.703702	3.302386	3.139018	6.129283
mmu-miR-6960-5p	mmu-mir-6960	2.410145	3.194684	2.727863	2.133517	3.147408	3.847253	1.626155	1.790259
mmu-miR-6963-3p	mmu-mir-6963	2.811618	3.648378	3.316262	3.707698	3.273945	3.584848	3.896718	3.060609
mmu-miR-6966-3p	mmu-mir-6966	3.137965	2.726793	2.57794	2.635465	1.587305	2.234186	1.814803	2.11073
mmu-miR-6994-3p	mmu-mir-6994	4.617813	3.213837	5.281369	4.605841	3.575886	3.354506	3.273782	5.208531
mmu-miR-700-3p	mmu-mir-700	16.83904	20.23343	16.00079	15.00507	16.08481	21.32469	14.52699	13.62667
mmu-miR-7015-3p	mmu-mir-7015	5.211256	5.368464	3.515565	3.952157	3.573093	7.894596	5.108347	6.550793
mmu-miR-704	mmu-mir-704	26.70036	29.99001	27.61871	29.85149	26.40254	29.24273	26.22591	31.64569
mmu-miR-7043-3p	mmu-mir-7043	82.26452	85.58239	89.9578	89.98767	85.4679	81.81281	92.71839	105.7874
mmu-miR-7059-5p	mmu-mir-7059	7.480823	7.221471	8.264599	6.449479	4.465533	7.76204	10.61824	8.512126
mmu-miR-7062-5p	mmu-mir-7062	3.994767	2.98673	2.606229	4.258424	3.532953	2.923678	3.864891	4.654005
mmu-miR-7118-3p	mmu-mir-7118	5.006154	6.440619	5.090311	4.9849	3.680225	6.060309	4.899816	5.678517
mmu-miR-7219-3p	mmu-mir-7219	3.700127	3.59078	4.838583	2.347096	2.848817	2.901017	2.69025	2.773165
mmu-miR-744-5p	mmu-mir-744	942.7291	1031.054	943.9079	1018.565	959.1833	1041.548	920.4132	1011.248
mmu-miR-744-3p	mmu-mir-744	2.809133	3.758693	2.688582	2.998645	2.061274	2.226253	3.977477	3.616579
mmu-miR-760-3p	mmu-mir-760	14.00885	11.54496	12.03167	11.03442	9.671821	11.43452	7.433245	10.25814
mmu-miR-7648-3p	mmu-mir-7648	2.052327	3.431107	3.639053	2.277304	3.930158	3.93489	4.59192	4.303975
mmu-miR-7667-5p	mmu-mir-7667	1.965795	1.80197	3.347525	3.717681	3.448418	3.666768	3.591117	4.550943
mmu-miR-7670-3p	mmu-mir-7670	3.001293	2.510469	1.597541	3.420328	2.747062	2.660876	3.342267	2.551157
mmu-miR-7676-3p	mmu-mir-7676-1	3.897161	1.842223	3.624574	3.132788	3.714057	2.383345	3.190487	3.560036
mmu-miR-7676-3p	mmu-mir-7676-2	3.897161	1.842223	3.624574	3.132788	3.714057	2.383345	3.190487	3.560036
mmu-miR-7679-3p	mmu-mir-7679	1.653007	2.746122	1.714195	2.439818	1.828909	2.007243	1.40556	2.642935
mmu-miR-7a-5p	mmu-mir-7a-1	13701.5	14267.14	14732.04	16243.91	17088.7	16280.07	20546.7	21042.56
mmu-miR-7a-1-3p	mmu-mir-7a-1	2.409387	2.942078	2.835681	3.347367	3.751055	3.810601	3.282395	3.223617
mmu-miR-7a-5p	mmu-mir-7a-2	13620.09	14174.47	14633.08	16147.52	16980.69	16172.05	20414.31	20910.73
mmu-miR-7b-5p	mmu-mir-7b	57.63984	57.23619	61.65466	73.66534	75.362	68.67952	87.18033	85.42391
mmu-miR-8103	mmu-mir-8103	21.71162	15.96021	18.61957	14.53587	13.7039	17.62959	16.69786	13.88373

mmu-miR-8112	mmu-mir-8112	38.42885	41.48476	40.13236	44.71135	40.22427	43.78716	47.39335	39.92238
mmu-miR-8114	mmu-mir-8114	12.07341	14.44704	11.24411	15.63659	12.52713	14.15845	15.31365	15.492
mmu-miR-872-5p	mmu-mir-872	253.4521	251.5837	256.0587	239.6966	256.7305	232.4715	224.5318	242.1759
mmu-miR-872-3p	mmu-mir-872	9.178669	7.601309	11.58666	11.54549	9.335511	9.055705	8.774356	7.864129
mmu-miR-877-5p	mmu-mir-877	16.30596	13.881	18.91489	17.27659	17.91452	16.11924	13.35135	14.3336
mmu-miR-877-3p	mmu-mir-877	21.27477	22.55325	23.16893	26.4557	22.26869	21.89114	19.88547	18.82101
mmu-miR-9-5p	mmu-mir-9-1	2620.832	2580.548	2446.865	2188.046	2184.857	2292.826	2018.009	1985.942
mmu-miR-9-3p	mmu-mir-9-1	71.35824	69.6284	69.63047	63.54606	69.91763	65.1259	57.79985	55.57778
mmu-miR-9-5p	mmu-mir-9-2	2620.832	2580.548	2446.865	2188.046	2184.857	2292.92	2018.009	1985.942
mmu-miR-9-3p	mmu-mir-9-2	71.35824	69.6284	69.63047	63.54606	69.91763	65.1259	57.79985	55.57778
mmu-miR-9-5p	mmu-mir-9-3	2615.799	2577.392	2443.537	2185.769	2182.594	2291.264	2015.009	1983.602
mmu-miR-9-3p	mmu-mir-9-3	71.24511	69.07612	68.83594	63.22684	69.50286	65.40343	57.07052	55.78391
mmu-miR-92a-3p	mmu-mir-92a-1	9509.44	9018.104	9114.413	9281.871	9630.478	9798.226	10041.22	9750.184
mmu-miR-92a-1-5p	mmu-mir-92a-1	18.9961	22.64148	23.84896	26.14237	21.71649	16.58105	17.42111	19.50911
mmu-miR-92a-3p	mmu-mir-92a-2	1529.083	1030.955	997.0273	1053.842	1057.815	1123.112	1332.439	1407.159
mmu-miR-92b-3p	mmu-mir-92b	17.82445	14.79163	18.81219	18.00671	14.4998	21.47153	17.2068	14.80638
mmu-miR-93-5p	mmu-mir-93	316.0639	304.4275	316.0299	299.7209	315.28	321.4498	266.6759	268.2659
mmu-miR-93-3p	mmu-mir-93	4.217545	4.057205	4.617598	4.080487	3.841525	3.9337	6.919819	3.05739
mmu-miR-935	mmu-mir-935	9.780845	12.29561	8.594139	12.24824	12.36748	11.54549	9.468193	10.71855
mmu-miR-96-5p	mmu-mir-96	326.2852	312.7454	338.6968	307.5293	323.7914	346.9965	322.1414	339.4989
mmu-miR-98-5p	mmu-mir-98	1843.028	1881.232	1871.705	1835.864	1860.929	1798.616	1855.677	1781.61
mmu-miR-98-3p	mmu-mir-98	6.030814	6.807726	6.92116	6.88511	5.846953	5.992721	5.110178	3.863796
mmu-miR-99a-5p	mmu-mir-99a	86300.31	88310.29	89829.8	85429.77	84581.42	85199.79	82181.3	86263.49
mmu-miR-99b-5p	mmu-mir-99b	12780.92	12346.85	12185.68	12905.74	12709.89	13155.46	13998.18	15303.69
mmu-miR-99b-3p	mmu-mir-99b	428.2971	402.1953	441.8715	536.7536	551.1126	520.1822	600.8698	707.334

Table S6. DEM comparing LPS 3 h and 8 h.

miRNA	precursor	logFC	logCPM	F	PValue	FDR	color
mmu-miR-146b-5p	mmu-mir-146b	-0.60628	15.10582	175.205	8.27E-13	3.08E-10	Down

mmu-miR-155-5p	mmu-mir-155	-0.70408	7.851463	52.41137	1.36E-07	1.27E-05	Down
mmu-miR-146a-5p	mmu-mir-146a	-0.51983	13.44443	37.74123	2.00E-06	0.000123	Down
mmu-miR-7a-5p	mmu-mir-7a-1	-0.33894	14.03084	37.34326	2.16E-06	0.000123	Down
mmu-miR-7a-5p	mmu-mir-7a-2	-0.3382	14.02166	37.03755	2.31E-06	0.000123	Down
mmu-miR-146b-3p	mmu-mir-146b	-0.49436	5.335017	21.06993	0.000107	0.00363	Down
mmu-miR-671-3p	mmu-mir-671	-0.48954	7.899618	20.70425	0.000119	0.003691	Down
mmu-miR-365-3p	mmu-mir-365-1	-1.45755	3.072524	18.50745	0.000227	0.006284	Down
mmu-miR-365-3p	mmu-mir-365-2	-1.45755	3.076104	18.3761	0.000237	0.006284	Down
mmu-miR-664-3p	mmu-mir-664	-0.73219	3.803318	10.07834	0.003949	0.048969	Down
mmu-miR-155-3p	mmu-mir-155	1.505826	4.551287	94.40255	5.94E-10	1.10E-07	Up
mmu-miR-132-5p	mmu-mir-132	0.75471	5.783253	35.89421	2.93E-06	0.000136	Up
mmu-miR-365-2-5p	mmu-mir-365-2	0.924483	4.241474	14.98586	0.000688	0.014211	Up
mmu-miR-139-3p	mmu-mir-139	0.402193	8.159731	11.28489	0.002504	0.035135	Up
mmu-miR-455-5p	mmu-mir-455	1.245696	1.772278	10.68212	0.003175	0.04073	Up

Table S7. List of miRNet2.0 mapping for 11 T-DEMs (149 were unique identified genes)

Target	ID	logFC	logCPM	F	PValue	FDR
Abhd16a	mmu-miR-155-5p	-0.13745	5.184402	8.291411	0.00732	0.010103
Ado	mmu-miR-664-3p	0.047504	6.047136	1.626027	0.212142	0.243329
Agtrap	mmu-miR-155-5p	-0.18458	5.802254	30.6248	5.34E-06	1.06E-05
Akt1	mmu-miR-155-5p	-0.12814	8.584205	25.10378	2.32E-05	4.29E-05
Aldh1l2	mmu-miR-664-3p	0.723479	2.920187	76.90557	9.70E-10	2.98E-09
Arid4a	mmu-miR-155-5p	0.941864	5.082409	391.8789	1.18E-18	1.60E-17
Arid4b	mmu-miR-664-3p	0.188642	5.472106	28.71794	8.72E-06	1.69E-05
Arntl	mmu-miR-155-5p	0.150953	5.079914	13.93253	0.000801	0.001243
Arpc3	mmu-miR-132-5p	0.042892	7.71525	2.317103	0.138539	0.16354
Atl2	mmu-miR-664-3p	-0.17847	5.788415	18.28452	0.000181	0.000302
Atp2a2	mmu-miR-664-3p	-0.43253	9.801772	235.0263	1.20E-15	8.95E-15

Bach1	mmu-miR-155-5p	-0.0785	6.022555	6.938901	0.013265	0.017757
Brwd1	mmu-miR-155-5p	0.192038	7.026641	31.32147	4.48E-06	8.91E-06
Btg2	mmu-miR-132-5p	-0.65685	5.293566	237.4969	1.05E-15	7.88E-15
Calr	mmu-miR-455-5p	-0.66439	10.11696	619.694	1.91E-21	4.44E-20
Camk2a	mmu-miR-146a-5p | mmu-miR-146b-5p	-0.10355	2.601511	0.788716	0.381627	0.419138
Camk2d	mmu-miR-146a-5p	-1.00528	5.559772	479.9948	7.00E-20	1.19E-18
Camta1	mmu-miR-155-5p	-0.41614	2.828018	14.31745	0.000698	0.001091
Ccnd2	mmu-miR-132-5p	0.337732	6.840856	114.7424	1.02E-11	4.05E-11
Ccnh	mmu-miR-155-5p	0.298439	4.35431	20.67003	8.53E-05	0.000148
Cdc27	mmu-miR-664-3p	-0.38545	7.031442	168.2241	9.21E-14	4.90E-13
Cdk6	mmu-miR-132-5p	-0.15842	9.151627	27.08958	1.34E-05	2.55E-05
Cebpb	mmu-miR-155-5p	-0.03553	8.104799	0.685797	0.414205	0.452169
Cpd	mmu-miR-155-5p | mmu-miR-365-3p	0.188616	9.583476	45.4669	1.88E-07	4.41E-07
Creb1	mmu-miR-7a-5p	0.197706	6.144429	20.41282	9.23E-05	0.000159
Cry2	mmu-miR-7a-5p	0.599202	4.292637	88.74254	2.00E-10	6.67E-10
Csnk1a1	mmu-miR-155-5p	-0.32751	8.714455	146.0889	5.42E-13	2.54E-12
Csnk1g2	mmu-miR-155-5p	-0.34551	6.626288	145.3833	5.75E-13	2.69E-12
Dcaf10	mmu-miR-7a-5p	-0.10995	4.990747	5.960537	0.02081	0.027305
Ddx27	mmu-miR-7a-5p	-0.60788	6.372825	323.7927	1.62E-17	1.74E-16
Dnajb1	mmu-miR-155-3p	-0.2514	5.505325	31.96842	3.81E-06	7.64E-06
Dnajb2	mmu-miR-155-3p	1.114802	4.00302	273.0122	1.63E-16	1.44E-15
Dusp16	mmu-miR-146a-5p	-0.5275	7.727684	311.632	2.73E-17	2.80E-16
Eif4e	mmu-miR-7a-5p	-0.56346	7.230928	445.8876	1.96E-19	3.03E-18
Ep300	mmu-miR-132-5p	0.330513	7.43952	157.952	2.04E-13	1.03E-12
Etv3	mmu-miR-155-5p	-0.36701	7.449175	170.4249	7.81E-14	4.21E-13
Fadd	mmu-miR-155-5p	0.105674	3.239471	1.163441	0.289438	0.324285
Fam160b2	mmu-miR-664-3p	0.292471	4.492449	29.35771	7.38E-06	1.44E-05
Fat3	mmu-miR-7a-5p	1.048375	0.984754	35.47059	1.64E-06	3.44E-06
Fos	mmu-miR-155-5p	0.386599	3.90711	32.18849	3.61E-06	7.26E-06
Gla	mmu-miR-7a-5p	-0.67181	5.387875	141.3987	8.11E-13	3.73E-12

Glul	mmu-miR-155-5p	-0.92875	3.686586	123.0131	4.44E-12	1.84E-11
Gmeb1	mmu-miR-7a-5p	-0.79449	4.958277	285.687	8.86E-17	8.30E-16
Gpalpp1	mmu-miR-664-3p	-0.11906	5.327438	5.775011	0.022715	0.029706
Gpr157	mmu-miR-146a-5p | mmu-miR-146b-5p	0.957495	0.928899	17.85412	0.000208	0.000345
Gpr65	mmu-miR-155-5p	0.77278	3.148044	71.95366	1.98E-09	5.87E-09
Gsk3b	mmu-miR-155-5p	0.57049	6.965092	336.8534	9.45E-18	1.06E-16
Hbp1	mmu-miR-155-5p	1.03546	4.028003	211.3496	4.87E-15	3.22E-14
Hdac4	mmu-miR-155-5p | mmu-miR-132-5p	0.703427	4.770877	166.3557	1.06E-13	5.59E-13
Hdac7	mmu-miR-7a-5p	1.706871	1.220957	88.64881	2.02E-10	6.75E-10
Hells	mmu-miR-7a-5p	-0.68704	6.825506	338.4602	8.85E-18	1.01E-16
Herpud2	mmu-miR-7a-5p	0.377576	5.672228	107.8672	2.12E-11	8.02E-11
Ikbke	mmu-miR-155-5p	-0.77016	7.524963	699.8587	3.38E-22	9.53E-21
Il6	mmu-miR-146a-5p	0.836057	2.448436	88.64574	8.22E-10	2.54E-09
Il6ra	mmu-miR-155-5p	1.367759	4.195117	343.9341	7.11E-18	8.20E-17
Inpp5d	mmu-miR-155-5p	1.137834	7.476875	1507.315	5.25E-27	5.22E-25
Irak2	mmu-miR-146a-5p	-0.36924	7.126781	244.2852	7.21E-16	5.56E-15
Isoc1	mmu-miR-155-5p	-0.65293	5.562153	157.9409	2.04E-13	1.03E-12
Jarid2	mmu-miR-155-5p	-1.23726	5.129633	721.4864	2.19E-22	6.51E-21
Klf4	mmu-miR-7a-5p	-0.24042	2.414266	4.034372	0.053753	0.067134
Kras	mmu-miR-155-5p	-0.34833	6.44749	90.92364	1.52E-10	5.15E-10
Lcor	mmu-miR-155-5p	0.10895	6.188711	11.66268	0.001865	0.00277
Lpin1	mmu-miR-155-5p	0.900899	5.612228	460.8393	1.24E-19	1.99E-18
Mafb	mmu-miR-155-5p	-0.59259	2.10892	34.65974	1.99E-06	4.12E-06
Map1b	mmu-miR-146a-5p	-0.77865	5.299115	286.0554	8.71E-17	8.16E-16
Mapk1	mmu-miR-155-5p	0.064271	7.662961	7.509474	0.010284	0.013967
Mapkap1	mmu-miR-7a-5p	0.561673	5.295982	119.6798	6.17E-12	2.53E-11
Mbp	mmu-miR-7a-5p	1.07023	6.159778	740.7188	1.50E-22	4.67E-21
Mecp2	mmu-miR-132-5p	0.130847	5.517062	7.167727	0.01197	0.016087
Med1	mmu-miR-146a-5p	-0.39269	7.310297	223.2917	2.37E-15	1.66E-14
Mef2a	mmu-miR-155-5p	-0.37057	7.566445	119.6503	6.19E-12	2.53E-11

Mfsd6	mmu-miR-155-5p | mmu-miR-365-3p	1.042639	6.857794	886.0178	1.15E-23	4.88E-22
Milr1	mmu-miR-7a-5p	0.778623	6.348582	556.3562	8.78E-21	1.75E-19
Mitf	mmu-miR-155-3p	-1.56935	8.469747	2997.573	2.23E-31	1.26E-28
Mknk1	mmu-miR-7a-5p	0.675583	5.397475	283.8571	9.67E-17	8.97E-16
Mknk2	mmu-miR-7a-5p	-0.22535	6.154687	45.54052	1.85E-07	4.35E-07
Mllt3	mmu-miR-146a-5p | mmu-miR-146b-5p	1.266659	3.5798	213.0589	4.38E-15	2.93E-14
Mr1	mmu-miR-155-5p	0.233238	1.524107	1.354462	0.253767	0.28737
Mtf1	mmu-miR-139-3p	0.068007	5.752117	4.0877	0.052289	0.065389
Mtfmt	mmu-miR-7a-5p	-0.22531	4.23317	12.77828	0.001223	0.001858
Mtpn	mmu-miR-132-5p	-0.12989	9.941386	26.4264	1.61E-05	3.02E-05
Nav2	mmu-miR-7a-5p | mmu-miR-139-3p	-0.66267	5.686541	123.1497	4.38E-12	1.82E-11
Nfkb1	mmu-miR-146b-5p	-1.20983	9.03143	1869.371	2.27E-28	3.59E-26
Nlrp3	mmu-miR-7a-5p	-2.00273	9.965827	3552.215	1.83E-32	1.65E-29
Nos2	mmu-miR-146a-5p	1.630039	4.498732	352.5691	5.06E-18	6.01E-17
Nr1h3	mmu-miR-155-5p	1.288545	2.722486	136.2007	1.29E-12	5.69E-12
Nr2c1	mmu-miR-664-3p	-0.20768	3.892161	6.484595	0.016313	0.021651
Nras	mmu-miR-132-5p	0.068234	7.87964	7.436988	0.010619	0.014391
Nup133	mmu-miR-7a-5p	-0.799	4.693386	161.8972	1.50E-13	7.73E-13
Ogdh	mmu-miR-664-3p	-0.23029	7.658115	90.99988	1.50E-10	5.11E-10
Opa1	mmu-miR-155-5p	-0.07405	6.70841	7.145833	0.012087	0.016238
Opa3	mmu-miR-139-3p	-0.73121	6.043482	537.9542	1.41E-20	2.71E-19
Otud6b	mmu-miR-664-3p	-0.28158	5.929724	75.28085	1.22E-09	3.71E-09
Paip2	mmu-miR-132-5p	-0.10225	5.695806	7.229796	0.011642	0.015687
Parp1	mmu-miR-7a-5p	-0.403	7.072835	205.0394	7.23E-15	4.62E-14
Pax6	mmu-miR-7a-5p	1.34087	3.654004	200.1333	9.91E-15	6.20E-14
Pea15a	mmu-miR-155-5p	0.079983	6.79546	6.136011	0.019169	0.025244
Pias3	mmu-miR-155-5p	0.780359	3.851401	112.2351	1.33E-11	5.15E-11
Pik3r1	mmu-miR-132-5p	1.112481	5.565654	686.2349	4.47E-22	1.24E-20
Pmaip1	mmu-miR-155-5p	0.815376	3.761737	120.5011	5.69E-12	2.34E-11

Polr2l	mmu-miR-7a-5p	-0.23719	1.869724	2.831845	0.102901	0.123695
Pramef8	mmu-miR-365-3p	-0.09428	4.964195	5.434547	0.026726	0.034669
Pten	mmu-miR-365-3p	0.369104	7.071545	137.0985	1.19E-12	5.29E-12
Ptk2	mmu-miR-7a-5p	0.540256	6.756737	309.6227	2.98E-17	3.03E-16
Ptprj	mmu-miR-155-5p	-1.26349	8.617801	1000.144	2.01E-24	1.02E-22
Rc3h1	mmu-miR-146a-5p	-0.66058	7.955982	544.5256	1.19E-20	2.31E-19
Rcbtb2	mmu-miR-155-5p	-0.56729	3.268832	43.33884	2.91E-07	6.70E-07
Relb	mmu-miR-146a-5p	-1.56507	5.81347	1875.876	2.16E-28	3.47E-26
Rheb	mmu-miR-155-5p	-0.43008	6.582512	196.5127	1.26E-14	7.68E-14
Rhoa	mmu-miR-155-5p	-0.04104	8.064927	2.882616	0.1	0.120409
Rhod	mmu-miR-155-5p	2.846623	1.735771	221.7013	2.60E-15	1.81E-14
Rictor	mmu-miR-155-5p	-0.38104	6.558151	157.7847	2.07E-13	1.04E-12
Ripk1	mmu-miR-155-5p	0.230456	6.556086	58.73293	1.62E-08	4.27E-08
Rnf6	mmu-miR-155-5p	-0.18496	6.620416	44.4707	2.30E-07	5.35E-07
Rnmt	mmu-miR-664-3p	-0.04493	6.191522	1.42844	0.241479	0.274465
Rps6kb1	mmu-miR-7a-5p	-0.27574	6.156115	73.35462	1.61E-09	4.83E-09
Runx2	mmu-miR-132-5p	0.529002	4.566209	78.23368	8.05E-10	2.50E-09
S1pr1	mmu-miR-155-5p	2.044741	3.443255	264.8971	2.45E-16	2.08E-15
Sco1	mmu-miR-139-3p	-0.06559	4.303054	1.167323	0.28865	0.32347
Scoc	mmu-miR-664-3p	0.088393	3.446107	1.208333	0.280496	0.315152
Sdcbp	mmu-miR-155-5p	-0.28686	8.987454	89.62864	1.79E-10	6.00E-10
Sgk3	mmu-miR-155-5p | mmu-miR-146a-5p | mmu-miR-146b-5p	0.639942	6.055026	393.8795	1.10E-18	1.49E-17
Sike1	mmu-miR-7a-5p	0.697853	5.653054	337.1951	9.32E-18	1.05E-16
Sirt1	mmu-miR-146b-5p	-0.4617	4.849897	85.76355	2.93E-10	9.59E-10
Ski	mmu-miR-155-5p	0.629102	6.640829	206.2635	6.69E-15	4.30E-14
Slc12a2	mmu-miR-664-3p	-0.29018	5.568944	42.3306	3.59E-07	8.18E-07
Smad4	mmu-miR-146b-5p | mmu-miR-664-3p	-0.1551	6.384632	22.85478	4.43E-05	7.92E-05
Smarca5	mmu-miR-132-5p	-0.51994	6.981933	400.963	8.58E-19	1.19E-17
Sp1	mmu-miR-7a-5p	-0.76823	7.497676	824.558	3.24E-23	1.17E-21
Stat1	mmu-miR-146a-5p	0.394525	2.908163	18.94887	0.000146	0.000246

Stxbp2	mmu-miR-7a-5p	0.090054	6.188245	8.594229	0.006433	0.008929
Tab2	mmu-miR-155-5p	-0.16869	7.578223	46.76488	1.45E-07	3.44E-07
Tcf7l2	mmu-miR-155-5p	0.55631	3.059203	37.64959	9.96E-07	2.15E-06
Tgif1	mmu-miR-146a-5p	0.189834	6.233501	18.79847	0.000153	0.000258
Tle4	mmu-miR-155-5p	0.112514	5.46604	7.725927	0.009351	0.012769
Tnf	mmu-miR-132-5p	-1.7852	12.35829	2671.366	1.21E-30	4.83E-28
Trappc13	mmu-miR-7a-5p	0.263564	5.537803	49.09965	9.22E-08	2.24E-07
Trim12c	mmu-miR-664-3p	0.316369	2.404087	7.936953	0.009842	0.0134
Trp53inp1	mmu-miR-155-5p	1.697792	4.192793	334.8586	1.02E-17	1.15E-16
Tspan14	mmu-miR-155-5p	1.215659	5.389905	585.0256	4.31E-21	9.16E-20
Vegfa	mmu-miR-146a-5p	-0.6678	6.568151	247.5748	6.04E-16	4.72E-15
Wbp1l	mmu-miR-155-5p	0.541175	6.264287	275.6128	1.44E-16	1.28E-15
Wdr59	mmu-miR-664-3p	-0.22907	4.600149	13.25789	0.001024	0.001571
Zfp236	mmu-miR-155-5p	-0.29516	5.946606	69.06724	3.06E-09	8.82E-09
Zfp26	mmu-miR-7a-5p	-0.4644	5.824738	114.211	1.08E-11	4.26E-11
Zfp322a	mmu-miR-155-5p	-0.28473	3.979325	14.91634	0.000564	0.000893
Zfp62	mmu-miR-664-3p	-0.14886	5.767401	14.93989	0.000559	0.000886
Zfp652	mmu-miR-155-5p	0.830676	5.453598	366.9487	2.92E-18	3.67E-17
Znrf3	mmu-miR-155-5p	-0.12784	4.273052	4.388229	0.044826	0.056575

Table S8. DEM for LPS 8h with ladostigil relative to LPS 8h.

miRNA	precursor	logFC	logCPM	F	PValue	FDR	color
mmu-miR-23b-5p	mmu-mir-23b	0.565567	6.111998	22.82504	6.60E-05	0.021563	Up
mmu-miR-27a-5p	mmu-mir-27a	0.349551	9.05569	20.79834	0.000116	0.021563	Up
mmu-miR-27b-5p	mmu-mir-27b	0.354759	7.38776	15.91699	0.000507	0.049128	Up
mmu-miR-365-2-5p	mmu-mir-365-2	0.955202	4.241474	15.10874	0.00066	0.049128	Up
